# Small-Molecule Inhibitors That Target Protein–Protein Interactions in the RAD51 Family of Recombinases

**DOI:** 10.1002/cmdc.201402428

**Published:** 2014-12-02

**Authors:** Duncan E Scott, Anthony G Coyne, Ashok Venkitaraman, Tom L Blundell, Chris Abell, Marko Hyvönen

**Affiliations:** [a] Department of Biochemistry, University of Cambridge80 Tennis Court Road, Old Addenbrooke's Site, Cambridge, CB2 1GA (UK) E-mail: mh256@cam.ac.uk; [b] Department of Chemistry, University of CambridgeLensfield Road, Cambridge, CB2 1EW (UK); [c] Medical Research Council Cancer Unit, University of CambridgeHills Road, Cambridge CB2 0XZ (UK)

**Keywords:** biophysics, BRCA2, homologous recombination, inhibitors, protein–protein interactions, RAD51

## Abstract

The development of small molecules that inhibit protein–protein interactions continues to be a challenge in chemical biology and drug discovery. Herein we report the development of indole-based fragments that bind in a shallow surface pocket of a humanised surrogate of RAD51. RAD51 is an ATP-dependent recombinase that plays a key role in the repair of double-strand DNA breaks. It both self-associates, forming filament structures with DNA, and interacts with the BRCA2 protein through a common “FxxA” tetrapeptide motif. We elaborated previously identified fragment hits that target the FxxA motif site and developed small-molecule inhibitors that are approximately 500-fold more potent than the initial fragments. The lead compounds were shown to compete with the BRCA2-derived Ac-FHTA-NH_2_ peptide and the self-association peptide of RAD51, but they had no effect on ATP binding. This study is the first reported elaboration of small-molecular-weight fragments against this challenging target.

## Introduction

Protein–protein interactions (PPIs) are ubiquitous in nature and play key roles in many cellular processes including apoptosis, signal transduction, gene expression, and DNA repair and replication. It is not surprising that the inhibition of certain PPIs has been hypothesised to have implications in many diseases, including cancer.[1, 2] PPIs offer many potential targets for small-molecule intervention in disease, but are a challenging and underrepresented target class in modern drug discovery. PPI sites bury greater surface areas than protein–ligand binding sites, and are generally featureless by comparison,[3] often lacking major concave binding pockets that naturally bind small molecules.[4] PPI inhibitors consequently tend to bind in many smaller pockets.[5] As a consequence of these inherent differences between PPI sites and traditional targets, PPI inhibitors tend to be larger, more hydrophobic, and form fewer hydrogen bonds than the small-molecule ligands of enzymes.[6]

Despite these challenges, there have been a number of successes in the development of PPI inhibitors, with various strategies showing success against a diverse set of targets.[1] High-throughput screening (HTS) is a common approach in modern drug discovery, and was used successfully to develop the Nutlin compounds, which bind MDM2 at the p53 binding site.[7] However, HTS methodology has enjoyed limited success against this target class.[8] For example, Fairbrother and co-workers reported the failure of HTS in targeting the interaction between the BIR3 domain of XIAP, and the partner protein Smac; in this case, however, a screen of structure-based libraries designed on the Smac binding epitope “AVPI” proved successful.[8]

Fragment-based drug discovery (FBDD) has met with some success in the field of PPI inhibitors. Briefly, in FBDD, low-molecular-weight compounds are screened at relatively high concentrations to identify weak, yet efficient binders.[9] Typically, structural information regarding the fragment–protein complex is then used to guide fragment “growth”—chemical elaboration of the original hit—in order to establish additional interactions. Different fragments may be found to bind simultaneously in adjacent pockets, in which case there may be an opportunity for fragment “linking” to greatly improve potency, as was reported by Petros et al. with a series of Bcl-2 inhibitors.[10] The interaction between the anti-apoptotic Bcl-2 and BH3 proteins is defined by a deep hydrophobic groove in Bcl-2 and has been successfully targeted with compounds that have shown antitumour activity in xenograft experiments,[11] and recently in patients.[12] Further compounds have now begun to show promise in clinical trials.[13, 14] A fragment screen against the bacterial membrane-anchored protein ZipA identified fragments that bind to the C-terminal region of ZipA. Compounds were then developed and were shown to block the binding of an α-helix peptide from FtsZ.[15] Fesik and colleagues recently used a fragment-based approach to develop small molecules that prevent Sos binding to K-Ras by binding at a surface site of K-Ras.[16] Despite these successes, identification of fragments at PPI sites is not always possible. For example, fragmented MDM2 compounds, which were screened as fragments, were not found to show detectable activity unless they filled two sub-pockets and were at the higher end of the typical molecular weight of fragments.[17] Also, Van Molle et al. reported being unable to detect the binding of fragments dissected from high-potency compounds against von Hippel–Lindau protein.[18]

**Figure 1:**
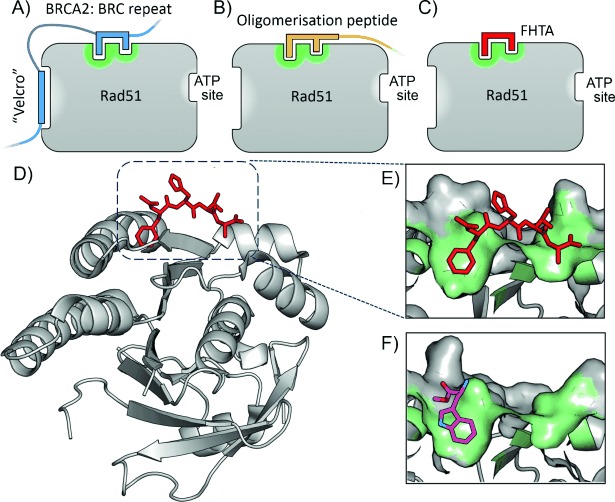
A)–C) Cartoon representation of RAD51 binding sites. In addition to an ATP binding site and a BRC repeat helix binding site (“Velcro” region), a surface binding pocket (green) binds the FxxA epitope of the BRC repeats of BRCA2 (A, blue) and the self-oligomerisation peptide of RAD51 (B, orange). The Ac-FHTA-NH_2_ tetrapeptide is indicated for clarity (C, red). D) Crystal structure of Ac-FHTA-NH_2_ (red) bound to RAD51 (PDB code 4B3B). E) Highlight showing surface pocket of RAD51; the contacting surface is represented in green. F) l-Methyl ester tryptophan 1 fragment previously identified that binds in the Phe pocket of HumRADA2 (PDB code 4B2I).[21]

In this work we targeted the protein–protein interaction between RAD51 and the tumour suppressor protein, BRCA2. RAD51 is a recombinase involved in the homology-directed repair of DNA double-strand breaks, forming an active filament with single-strand DNA in an ATP-hydrolysis-dependent process.[19] Structurally, its interaction with BRCA2 is mediated by eight BRC repeats that are characterised by a common FxxA motif (Figure 1 A).[20] In addition, the FxxA sequence motif occurs in the N-terminal linker region of RAD51, enabling it to self-oligomerise through interaction with this region to form RAD51 filaments (Figure 1 B). The FxxA oligomerisation motif of RAD51 is highly conserved across several species,[21] and six of the eight BRC repeats conserve the same motif across several mammalian BRCA2 proteins.[20] The structural basis of this FxxA motif was elucidated by Pellegrini et al., who solved the X-ray crystal structure of truncated human RAD51 in complex with the fourth BRC repeat (BRC4) from human BRCA2.[21] The structure revealed FxxA as part of a β-hairpin across the surface of RAD51, with Phe 1524 of BRC4 in a surface pocket on RAD51, and Ala 1527 contacting the surface in a much shallower pocket, across the central β-sheet of RAD51. As BRCA2 is involved in localisation of RAD51 and formation of a nucleoprotein filament on DNA at sites of double-strand breaks, we hypothesise that disrupting the interaction between RAD51 filament protomers and between RAD51 and BRCA2 is a valid strategy to increase the susceptibility of tumour cells to radiation and DNA-damaging agents.[22] Therefore, the development of a small molecule that disrupts these interactions by binding at the common FxxA binding site is of interest both as a chemical tool and as a potential therapeutic compound.

We previously published fragment-screening results using a humanised RAD51 surrogate from *Pyrococcus furiosus*, which we refer to as HumRADA2 (previously referred to as MAYSAM RadA).[23] In this surrogate protein, the N-terminal domain with the oligomerisation sequence was removed to monomerise the protein and expose the FxxA binding site for screening, and six surface residues were mutated around the Phe pocket in order to humanise the protein in this region. The tetrapeptide Ac-FHTA-NH_2_, derived from the BRC4 peptide, was found to have a *K*_D_ value of 250 μm, and the crystal structure in complex with humanised RadA was solved (Figure 1 D,E). A set of fragments that bind at the PPI interface in the Phe pocket of the FxxA motif were also identified, including indazole (*K*_D_=1000 μm), l-tryptophan methyl ester **1** (*K*_D_=570 μm, Figure 1 F), and 4-methyl ester indole (*K*_D_=1300 μm).

## Results and Discussion

Herein we present the development of these fragment hits from millimolar to low-micromolar potency. Initially, small modifications were made to **1**, which led to only small changes in potency (Table 1 compounds **2**–**4**). Compounds with more significant, yet still relatively modest modifications were then synthesised and tested by isothermal titration calorimetry (ITC) (Table 1). The benzyl ester **5 b** showed a small increase in potency, *K*_D_=200 μm, relative to the parent fragment **1**. Extending the carbon chain by another atom gave strikingly better potency: compound **5 c**, which at 35 μm, exhibited an approximate 10-fold improvement over the parent fragment **1**. An investigation of the length of the ester carbon chain revealed that the ethyl linker is optimal; both the phenyl ester **5 a** and the phenpropyl ester **5 d** showed poorer potency (Table 1). An SAR study focused around the phenyl group of compound **5 c** identified substituents that had only a moderate effect on potency and those that did not bind (*K*_D_>200 μm). Changing the ester group to an amide led to a large decrease in affinity (Table 1, compound **5 e**). A range of substituents are tolerated on the phenyl ring, with methyl, fluoro, and hydroxy groups (Table 1, compounds **5 f**–**j**), and no significant change in *K*_D_ was observed. However if 4-bromo and 4-methoxy groups were introduced to the phenyl ring, there was a significant decrease in affinity, presumably due to a steric clash with the protein at the *para* position (Table 1, compounds **5 k** and **5 l**). Replacing the phenyl ring with cyclohexyl or indole rings was also tolerated (Table 1, compounds **5 m** and **5 n**).

**Table 1:** SAR for tryptophan ester series binding to HumRADA2 (ITC)

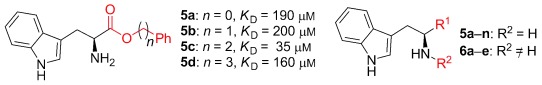					
Compd	R^1^	R^2^	LE^[a]^	LLE^[b]^	*K*_D_ [μm]^[c]^
**1**	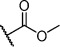	H	0.28	1.9	570[23]
**2**	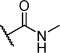	H	0.24	2.2	1500±24
**3**	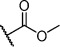	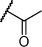	0.22	2.0	810±19
**4**	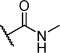	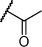	0.22	2.6	890±15
					
**5 a**	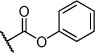	H	0.24	0.7	190±5
**5 b**	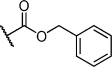	H	0.23	0.7	200±8
**5 c**	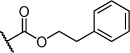	H	0.26	1.1	35±4
**5 d**	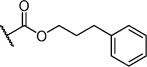	H	0.22	0.02	160±7
**5 e**	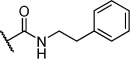	H	–	–	>200
**5 f**	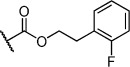	H	0.26	1.1	25±5
**5 g**	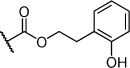	H	0.26	1.5	27±12
**5 h**	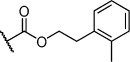	H	0.26	0.7	30±9
**5 i**	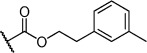	H	0.26	0.7	30±7
**5 j**	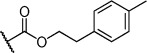	H	0.26	0.8	22±4
**5 k**	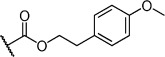	H	–	–	>200
**5 l**	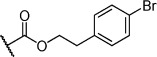	H	–	–	>200
**5 m**	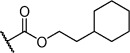	H	0.27	0.8	28±9
**5 n**	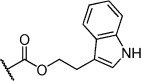	H	0.23	1.0	35±9
**6 a**	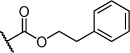	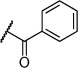	0.24	0.5	3±1
**6 b**	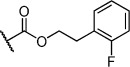	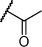	0.25	1.5	18±1
**6 c**	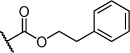	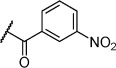	–	–	>200
**6 d**	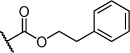	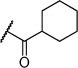	–	–	>200
**6 e**	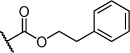	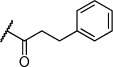	–	–	>200

[a] Ligand efficiency (kcal mol^−1^ per heavy atom). [b] LLE=p*K*_D_−clog *P*; clog *P* was calculated with Instant JChem (ChemAxon). [c] Estimated errors in *K*_D_ are given, generated by fitting a single-site binding model (Origin software version 7.0552).

Further modification of compound **5 c** also focused attention on the amino group. Modification with acetyl had no significant effect on affinity (Table 1, compound **6 b**). However, the introduction of benzoyl onto the amino group led to a significant increase in affinity: *K*_D_=3 μm (Table 1, compound **6 a**). A *meta*-nitrophenyl substituent, a cyclohexyl amide, and a phenethyl amide all failed to give detectable activity (Table 1, compounds **6 c**–**e**).

Inspired by the indole scaffold from tryptophan and other fragments previously reported to bind in this pocket,[23] we developed a series of sulfonamide derivatives of tryptamine. We reasoned that the indole motif would anchor in the Phe pocket, and a sulfonamide linkage with hydrogen bond acceptors and donors would have the ability to form hydrogen bonds to proximal side chains around the Phe pocket such as Gln 217 and the backbone of Tyr 202, to which the FHTA peptide also forms hydrogen bonds. Initially the phenyl sulfonamide **7 a** and the benzyl sulfonamide **7 b** were synthesised and tested by ITC. Interestingly, only the phenyl sulfonamide **7 a** exhibited evidence of binding, with a *K*_D_ value of 28 μm, indicating specific requirement for a directly attached phenyl ring. No activity was detectable for the benzyl substituent **7 b** or for the methyl sulfonamide compound **7 i**. The SAR around the phenyl ring was explored with compounds **7 c**–**h**, and the results are listed in Table 2. The introduction of a bromine atom at the 4-position of the phenyl ring led to a decrease in affinity to 92 μm; however, if the bromine substitution was at the 2-position, the affinity increased to 3.1 μm (Table 2, compounds **7 c** and **7 d**). The inclusion of NO_2_ or NH_2_ groups at the 3-position had no significant effect on affinity (Table 2, compounds **7 e** and **7 f**). Interestingly, if 4-methoxy was included on the phenyl ring, a decrease in affinity was observed, but if this was replaced with the structurally similar benzodihydrofuran (Table 2, compounds **7 g** and **7 h**) the affinity was recovered to 34 μm. Unsurprisingly, replacing the phenyl ring with a thiophene ring, which is sometimes used as a phenyl isostere, gave a *K*_D_ value similar to that of the phenyl group (Table 2, compound **7 j**). We then explored SAR around substituted thiophenes: compounds **7 k**–**p**. Clear improvements in potency were observed with benzothiophene (Table 2, compound **7 p**, *K*_D_=1.6 μm) and halogenated thiophenes, most notably thiophene **7 m**, which has a *K*_D_ value of 1.3 μm. We also explored a selection of more polar five-membered heterocycles, a substituted isoxazole **7 q**, a furan **7 r**, and a methylimidazole **7 s**. Whilst the furan possessed similar potency to the phenyl compound **7 a**, the isoxazole showed a drop in potency to 110 μm. The imidazole **7 s** was the amongst the weakest binding of all compounds (*K*_D_=420 μm). Taken together, the SAR seems largely driven by a hydrophobic interaction with the substituent on the sulfonamide; *para* substituents on phenyl rings are not well tolerated relative to *ortho* substituents, suggesting a steric clash or unfavourable interaction on the protein surface in a manner similar to that observed for the tryptophan ester series. Substituents on the thiophene scaffold yielded a number of potent compounds; however, it appears that hydrophobic groups appear to give the largest increases in potency. The more polar five-membered heterocycles showed decreased potency relative to the unsubstituted phenyl ring.

**Table 2:** SAR for substituted tryptamine series binding to HumRADA2 (ITC)

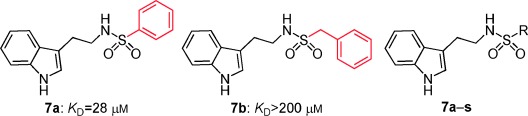				
Compd	R	LE^[a]^	LLE^[b]^	*K*_D_ [μm]^[c]^
**7 a**	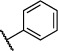	0.30	1.6	28±6
**7 b**	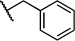	–	–	>200
**7 c**	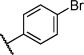	0.25	0.4	92±22
**7 d**	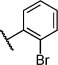	0.34	1.8	3.1±0.1
**7 e**	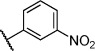	0.27	1.9	18±4
**7 f**	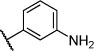	0.28	2.4	31±6
**7 g**	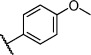	0.23	1.2	120±10
**7 h**	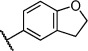	0.25	1.7	34±12
**7 i**	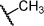	–	–	>200
**7 j**	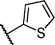	0.33	1.9	16±6
**7 k**	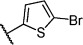	0.32	1.2	10±1
**7 l**	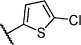	0.32	1.3	13±1
**7 m**	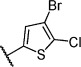	0.36	1.5	1.3±0.1
**7 n**	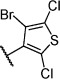	0.33	0.6	2.3±0.4
**7 o**	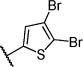	0.27	−0.4	67±29
**7 p**	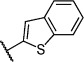	0.33	1.8	1.6±0.6
**7 q**	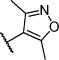	0.25	2.4	110±12
**7 r**	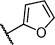	0.32	2.6	18±2
**7 s**	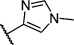	0.22	1.8	420±74

[a] Ligand efficiency (kcal mol^−1^ per heavy atom). [b] LLE=p*K*_D_−clog *P*; clog *P* was calculated with Instant JChem (ChemAxon). [c] Estimated errors in *K*_D_ are given, generated by fitting a single-site binding model (Origin software version 7.0552).

**Figure 2:**
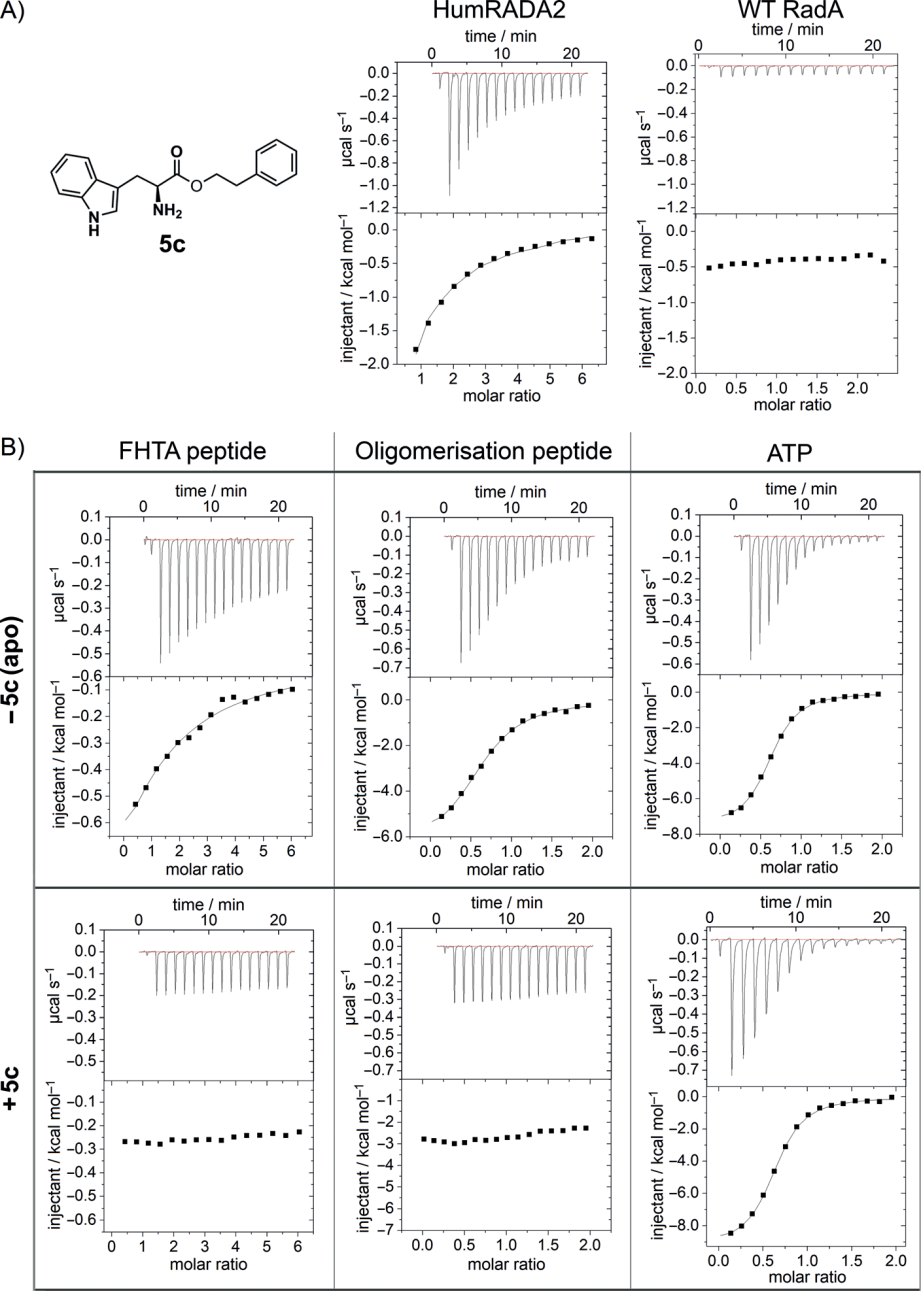
A) Structure of compound 5 c, and binding directly to HumRADA2 (left, *K*_D_=35 μm) and showing no binding to the wild-type RadA (right). B) Competitive ITC experiments to HumRADA2 indicate the binding site of 5 c. Top row: ITC experiments in the absence of 5 c determined the *K*_D_ values of Ac-FHTA-NH_2_, the RadA oligomerisation peptide, and ATP to be 290, 7.0, and 2.5 μm, respectively. Bottom row: ITC of Ac-FHTA-NH_2_, RadA oligomerisation peptide, and ATP in the presence of 200 μm 5 c shows competition with the FxxA binding site, but not the ATP binding site.

Unfortunately, attempts to crystallise and solve the structures of the more potent sulfonamide compounds and tryptophan ester compounds in complex with the target protein were unsuccessful. Often poor electron density for only a single ring was observed in the Phe pocket, but could not be unambiguously assigned to either ring of the molecule. It is possible that there is some specific molecular recognition in the pocket, but disorganisation in the binding modes as the molecule emerges from the Phe pocket, providing hydrophobic interactions with the protein surface. Therefore, in X-ray crystallography we might see part of a ligand bound in the Phe pocket, but a superposition of binding modes outside the pocket would render this part of the ligand invisible. Hence, the location of the binding site of compound **5 c** was investigated by ITC experiments (Figure 2). Binding of **5 c** could be detected directly against the HumRADA2 protein with a humanised FxxA binding site (*K*_D_=35 μm), but not against the unhumanised wild-type RadA protein (Figure 2 A). The importance of the humanising mutations around the Phe pocket to binding is highly suggestive of **5 c** interacting at this site. Competitive ITC experiments also implicated the FxxA binding site (Figure 2 B). Briefly, titrations of tetrapeptide Ac-FHTA-NH_2_ (*K*_D_=250 μm), the wild-type RadA oligomerisation peptide (*K*_D_=7.0 μm), and ATP (*K*_D_=2.5 μm) were performed individually against *apo* protein to measure their respective *K*_D_ values (top row titrations, Figure 2 B). The *K*_D_ measurement of each ligand was then repeated, but in the presence of 200 μm **5 c** (bottom row titrations, Figure 2 B). As expected for an FxxA site binder in this system, the binding of ATP was not affected by pre-incubation of the protein with **5 c**, but **5 c** competes with and blocks the binding of both the self-oligomerisation peptide and the Ac-FHTA-NH_2_ tetrapeptide. Taken together, these experiments provide strong evidence that the FHTA interaction site is the specific binding site of **5 c**. Analogous competitive ITC experiments with **7 m** also demonstrated blockage of the binding to humanised RAD51 of the oligomerisation peptide but not ATP, consistent with specific binding at the FHTA binding site (data not shown).

**Figure 3:**
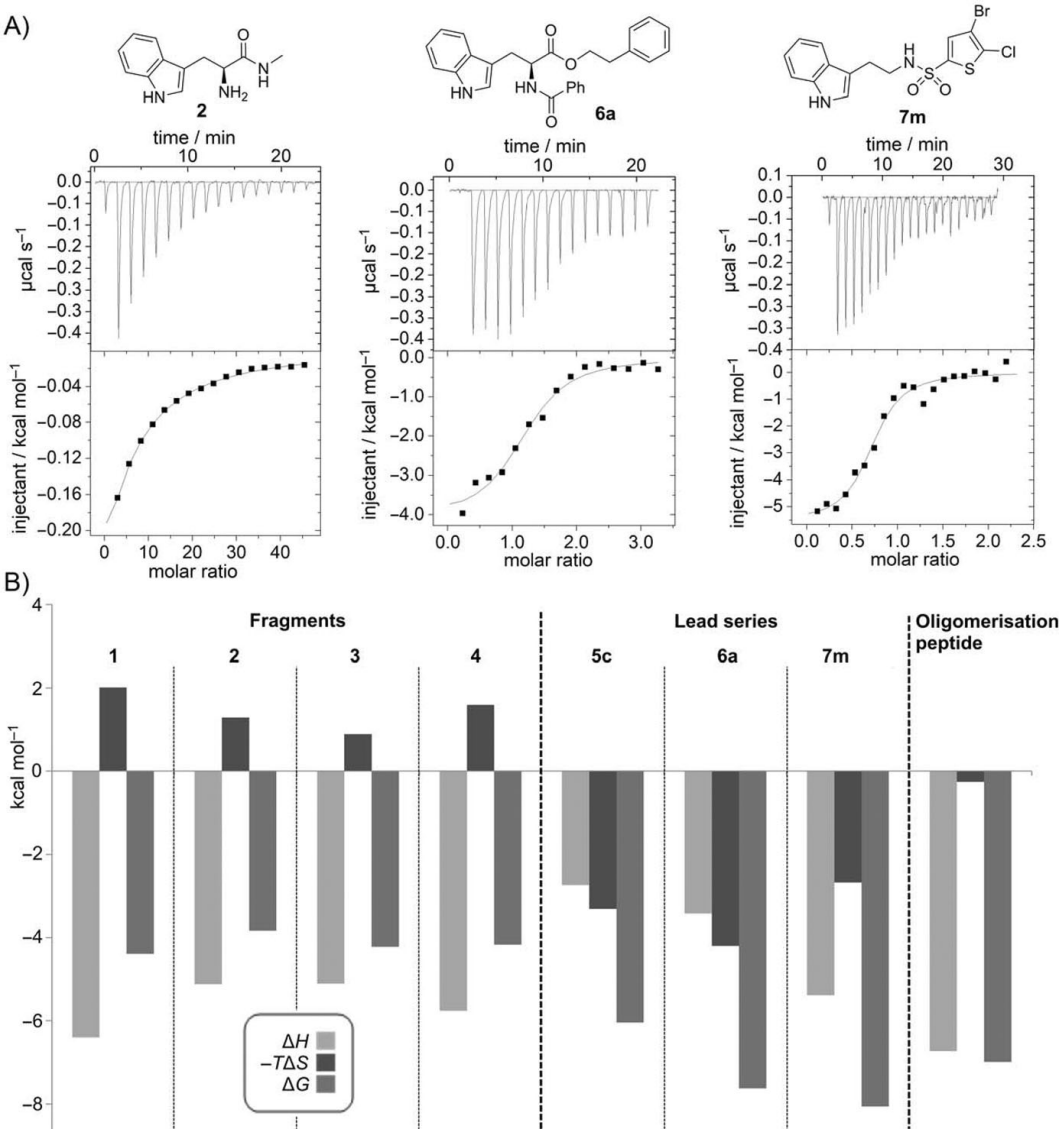
A) Direct ITC experiments with 2, 6 a, and 7 m against HumRADA2. B) Analysis of the thermodynamics of binding for various fragments and representative lead compounds 5 c (*K*_D_=35 μm), 6 a (*K*_D_=3 μm), and 7 m (*K*_D_=1.3 μm) to HumRADA2.

It has previously been observed with thrombin inhibitors that substituting a terminal cyclopentyl ring for a cyclohexyl group in the S3/S4 specificity pocket can have marked changes on the binding thermodynamics and the electron density observed in X-ray crystallography.[24] The binding of the cyclopentyl compound was enthalpically driven, and the cyclopentyl group was clearly observed bound to the protein by X-ray crystallography. In contrast, the cyclohexyl compound, which had the same *K*_D_ value, was driven to bind by favourable entropic factors; the cyclohexyl appendage was disordered and could not be observed crystallographically. For this reason, and the lack of clear electron density for our compounds, we analysed the entropic and enthalpic contributions to our developed series in greater detail. Comparison of the binding thermodynamics of the original fragment **1** and the modified fragments, compounds **2**–**4**, is shown in Figure 3 (Table S1 in Supporting Information). The binding of the fragments is enthalpically driven, overcoming an entropic penalty of binding of ∼2 kcal mol^−1^. The binding of the oligomerisation peptide, which binds with *K*_D_=7.0 μm, is almost entirely driven by favourable enthalpic contributions. However, for the representative lead series compounds **5 c**, **6 a**, and **7 m**, it is clear that increased potency has largely been achieved by optimisation of favourable entropic contributions. This switch in binding from enthalpy-driven fragments to an entropy-driven series is no doubt in part due to the hydrophobic nature of the attached groups. The favourable entropic component of binding of the lead series may reflect a disordered binding mode and may explain the difficulties in obtaining a crystal structure in complex with the protein. In retrospect, we can speculate that introducing hydrogen bond acceptors and donors to both rings might anchor both ends of the molecule, fixing a compound into an ordered binding mode. Although not necessarily imparting potency, presumably such a molecule would be more amenable to X-ray crystallography.

We have elaborated initial fragment hits that bind in a small surface pocket of a humanised RAD51 construct and have increased the potency by approximately 500-fold to ∼1 μm. Drug development is a multidimensional optimisation problem, however, and affinity for the target is not the only consideration. The utility of ligand efficiency metrics in drug discovery to assess the relative importance of affinity, log *P*, and molecular weight has recently been reviewed.[25] Ligand efficiency (LE) is defined as the free energy of binding in kcal mol^−1^ per non-hydrogen atom. An examination of the LEs of the compounds in each series reveals some interesting differences. In the tryptophan ester series, the starting fragment **1** (*K*_D_=570 μm) has an LE of 0.28. This value is slightly below the value of 0.3 kcal mol^−1^ per heavy atom, which is commonly accepted as a reasonable threshold for non-PPI targets; however, it has previously been noted that inhibitors of PPIs tend to have a lower LE of 0.24.[26] The developed phenethyl ester **5 c** (*K*_D_=35 μm) had an LE of 0.26, which fell subsequently to 0.24 for **6 a** (*K*_D_=3 μm), the most potent compound in this series. Conversely, several of the more potent compounds in the sulfonamide series possessed an improved LE, especially the most potent compound **7 m**, which had an LE of 0.36. This is a desirable achievement for any FBDD programme and even more impressive for small molecules that bind at a PPI site. Lipophilic ligand efficiency (LLE) is a ligand efficiency metric that penalises molecules which are highly lipophilic and is defined as the pIC_50_ (or p*K*_D_) minus clog *P*.[27] The higher the LLE value, the more potency is generated for a given lipophilicity. Although the potency of the compounds was not optimised with LLE in mind, it is interesting to retrospectively examine the LLEs. It has been suggested that optimised drug molecules should aim to possess an LLE of 5–7, although absolute values depend on the method used to calculate clog *P*. For the tryptophan series, the LLE generally drops as the compounds are progressed from fragment **1** (LLE=1.9), to **6 a** (LLE=0.5) for example. The situation is a little better for the sulfonamide series; the most potent compound **7 m** (*K*_D_=1.3 μm) has an LLE of 1.5. Compound **7 p**, which has a similar *K*_D_ value of 1.6 μm, is less lipophilic than **7 m** and has a marginally improved LLE of 1.8. Taken together, the metrics indicate that although the LE and LLE fall for the tryptophan series, the LLE can be maintained in the sulfonamide series whilst improving potency and actually increasing LE, suggesting the sulfonamide series is more attractive in terms of their suitability toward development.

## Conclusions

Although we were unable to obtain X-ray crystal structures for our best compounds bound to the protein, direct and competitive ITC experiments strongly implicate the FxxA binding region as the most probable binding site. A retrospective analysis of the thermodynamics of ligand binding reveals a strong entropic component, suggesting that the increase in log *P* of the compounds caused the potency to increase through hydrophobic interactions with the protein surface. With a lack of hydrogen bonds that might be thought to specifically orient a molecule, we hypothesise that a multitude of binding poses for the lipophilic moieties of the lead molecules may be superimposing in X-ray crystallography and causing a disappearance of ligand electron density. Therefore, a future strategy for elaboration of these compounds will involve introducing a balance of lipophilicity and polar interactions, as well as decreasing log *P*. We are currently developing functional assays with these compounds which will be reported at a later date.

## Supporting Information

As a service to our authors and readers, this journal provides supporting information supplied by the authors. Such materials are peer reviewed and may be re-organized for online delivery, but are not copy-edited or typeset. Technical support issues arising from supporting information (other than missing files) should be addressed to the authors.

miscellaneous_information
