# Supplementary material for: Small-Molecule Inhibitors That Target Protein–Protein Interactions in the RAD51 Family of Recombinases
Source: ChemMedChem. 2014 Dec 2;10(2):296–303. doi: 10.1002/cmdc.201402428 (PMC4506530; doi:10.1002/cmdc.201402428)
Supplement: Supplementary file 1 — miscellaneous_information [file cmdc0010-0296-sd1.pdf]

CHEMMEDCHEM
Chemistry & Drug Discovery

Supporting Information

© Copyright Wiley-VCH Verlag GmbH & Co. KGaA, 69451 Weinheim, 2015

Small-Molecule Inhibitors That Target Protein–Protein Interactions in the RAD51 Family of Recombinases

Duncan E. Scott,[b] Anthony G. Coyne,[b] Ashok Venkitaraman,[c] Tom L. Blundell,[a] Chris Abell,[b] and Marko Hyvönen*[a]

cmdc_201402428_sm_miscellaneous_information.pdf

Supplemental Data

Table S1: Thermodynamic binding profile of fragments, representative lead series compounds and oligomerisation peptide to HumRADA2.

Ligand	ΔH / kcal mol^{-1}	$-T\Delta S$ / kcal mol^{-1}	ΔG / kcal mol^{-1}
1	-6.4	2.0	-4.4
2	-5.1	1.3	-3.8
3	-5.1	0.9	-4.2
4	-5.8	1.6	-4.2
5c	-2.7	-3.3	-6.0
6a	-3.4	-4.2	-7.6
7m	-5.4	-2.7	-8.1
oligomerisation peptide	-6.7	-0.3	-7.0

Supplemental Experimental Procedures

Protein Preparation

C-terminal ATPase domain of *P. furiosus* RadA (residues 108–349, with deletion of the “L2” loop residues 288–314) was expressed in E.coli and purified as described before using ion-exchange ad size exclusion chromatographies.[1] Purified protein was concentrated to 0.5 mM, flash frozen in liquid nitrogen and stored at -80C. Protein concentration was determined by measuring absorbance at 280 nm in 6M GndHCl and using calculated absorption coefficient of 11,460 M^{-1} cm^{-1}

Isothermal Titration Calorimetry

ITC experiments were performed at 25°C on a MicroCal ITC-200 (GE Healthcare). HumRadA2 (humanized RadA) (600 μM in 20 mM MES pH 6.0 with 100 mM NaCl and 0.5 mM EDTA was diluted with Tris buffer (200 mM, pH 7.5 with 100 mM NaCl), and DMSO was added to match to the ligand solution. In titrations with ATP, 5 mM $MgCl_2$ was included in the ligand buffer and the protein buffer. Compounds in DMSO were diluted into the same Tris buffer to give a final concentration of 0.6–10 mM ligand in 10% DMSO and Tris buffer. Care was taken to ensure that the DMSO concentrations in the protein and ligand solutions were well matched so as to avoid artefacts arising from heats of dilution of DMSO. In a typical experiment, protein (60 μM) was loaded in the sample cell, and 16 injections (2.4 μL) of 4.8 s duration were made at 80 s intervals from a syringe loaded with ligand (0.6–10 mM) and rotating at 1000 rpm. In all titrations, an initial injection of ligand (0.4 μL) was made and discarded during data analysis. Control titrations of ligand to buffer were performed and showed insignificant heats. The thermodynamic parameters were obtained by fitting the data to a single-site-binding model by using Origin software and fixing the stoichiometry as 1.0 for weak binding ligands.

Compound Synthesis and Characterisation

Solvents and Reagents

THF was distilled under an atmosphere of dry nitrogen from lithium aluminum hydride and calcium hydride in the presence of triphenylmethane; DCM was distilled from calcium hydride. Reactions performed under an atmosphere of hydrogen chloride gas were maintained by an inflated balloon. N-Boc-tryptophan was purchased from Aldrich. Tryptamine was purchased from Acros. All other chemical were supplied from Aldrich, Alfa Aesar or Maybridge.

Caution: All reactions carried out using HCl gas were done in a fumehood with suitable personal protection equipment.

Chromatography

Thin layer chromatography (TLC) was performed on glass plates coated with Merck 60 F_{254} silica and visualization was achieved by UV light or by staining potassium permanganate. Flash column chromatography was carried out using Merck Kieselgel (230-400 mesh). Column chromatography was also carried out using a Biotage Isolera One and Biotage Isolera Four systems with UV detection at 254 nm and 280 nm.

Nuclear Magnetic Resonance Spectroscopy

NMR spectra were recorded on a Bruker Avance 400 (^{1}H: 400 MHz and ^{13}C: 100 MHz), or Bruker Avance Cryo 500 (^{1}H: 500 MHz and ^{13}C: 125 MHz). Chemical shifts are quoted in ppm and are referenced to the residual non-deuterated solvent peak, and are reported (based on appearance rather than interpretation) as follows: chemical shift δ/ppm (multiplicity, coupling constant J/Hz, number of protons) [br, broad; s, singlet; d, doublet; t, triplet; q, quartet; qui, quintet; sept, septet; m, multiplet]. All J values are given in Hz. In a number of ^{13}C NMR spectra there is overlap of the signals observed in the aromatic region. ^{1}H NMR, LCMS and HRMS confirm that the compounds are what are described.

Infrared Spectroscopy

Infrared spectra were recorded neat on a Perkin-Elmer Spectrum One spectrometer fitted with an attenuated total reflectance (ATR) attachment with internal referencing.

Mass Spectrometry

High-resolution mass measurements were performed on a Waters LCT Premier mass spectrometer or a Kratos Concept mass spectrometer. Low-resolution measurements were recorded on a Waters / ZQ LCMS[a] and on a Waters Aquity UPLC HClass LCMS[b].

Fragment elaboration

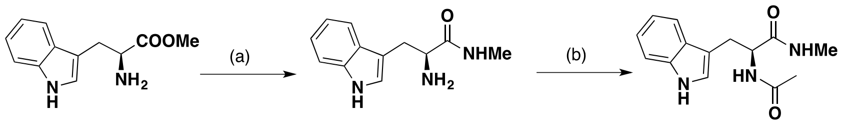

(a) $MeNH_2$ (8M THF soln), THF, RT, 15h. (b)Ac_2O, Pyridine,DCM 4ºC, 4h

Tryptophan ester series

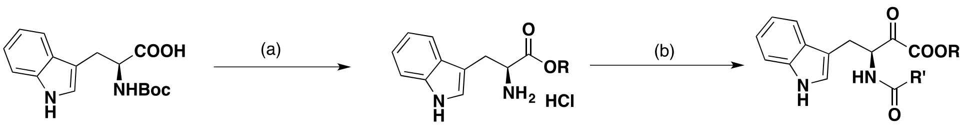

(a) 1. DCC, DMAP, **ROH or RNH_2**, 0-20ºC, 4-6h 2. HCl (g), Et_2O, RT, 1h (b) **R'COCl,** NEt_3, RT, 2-6 h

ROH or RNH_2

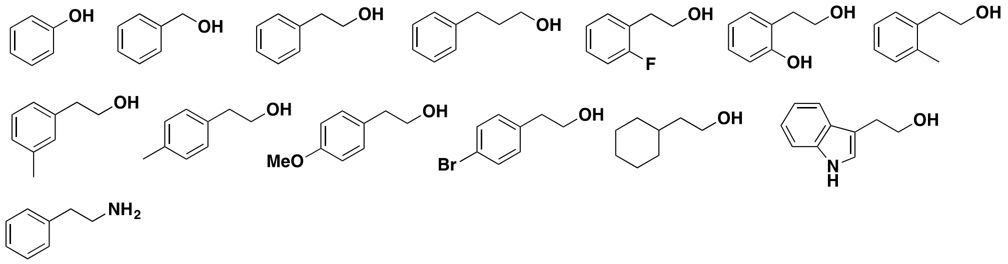

R'COCl

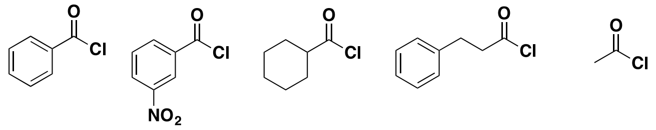

Indole sulfonamide series

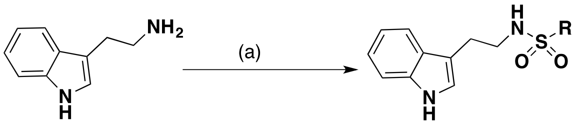

(a) **RSO_2Cl**, NEt_3, DCM, rt, 2-6 h

RSO_2Cl

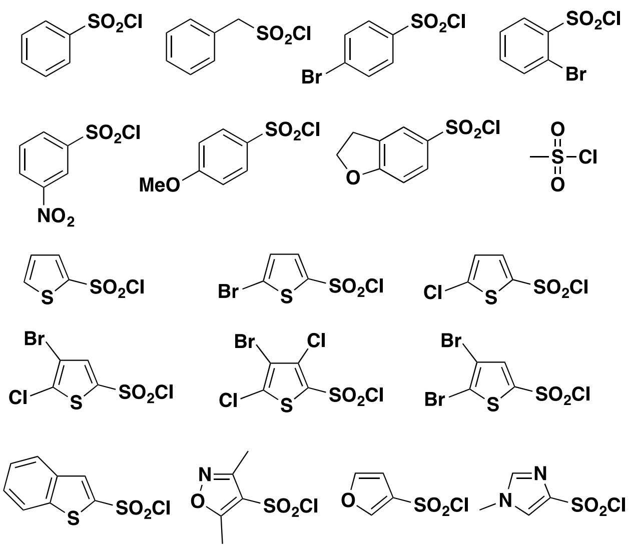

(*S*)-2-amino-3-(1H-indol-3-yl)-N-methylpropanamide (2)

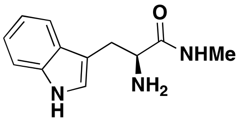

Methylamine (10 mL, 8.0 M in ethanol) was added to a suspension of L-tryptophan methyl ester hydrochloride (500 mg, 1.96 mmol) in THF (10 mL). The resulting solution was stirred overnight at room temperature and solvent removed *in vacuo* and the residue purified by silica chromatography to give the product as a white solid (392 mg, 92%).

^{1}H NMR (CD_3OD, 500 MHz) δ 2.67 (s, 3H), 3.13 (dd, *J* 7.4, 14.4, 1H,), 3.27 (ddd, *J* 0.6, 6.6, 14.5, 1H), 3.85 (t, *J* 7.0, 1H), 7.04 (ddd, *J* 1.0, 7.0, 8.0, 1H), 7.11 (ddd, *J* 1.1, 7.0, 8.1, 1H), 7.14 (s, 1H), 7.36 (td, *J* 8.2, 8.2, 1H), 7.59 (td, *J* 7.9, 0.9, 1H,). ^{13}C NMR (CD_3OD, 125 MHz) δ 26.3, 30.2, 55.9, 109.3, 112.5, 119.3, 119.9, 122.7, 125.1, 128.5, 138.4, 173.2. LCMS[a] calcd for $C_{12}H_{16}N_3O$ $(M+H)^+$ 218.1, found 218.1, R_t = 2.5 min HRMS: calcd for $C_{12}H_{16}N_3O$ $(M+H)^+$ 218.1288, found 218.1310;

(*S*)-methyl 2-acetamido-3-(1*H*-indol-3-yl)propanoate (3) [2]

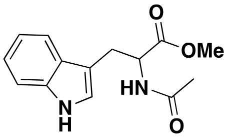

Acetic anhydride (0.075 mL, 0.79 mmol) was added dropwise to a solution of L-tryptophan methyl ester hydrochloride (165 mg, 0.65 mmol) in pyridine (4 mL) and DCM (10 mL). After stirring for two hours at room temperature, water was added (20 mL) and extracted with DCM (20 mL). The organic layer was dried *in vacuo* and the residue purified by silica chromatography (5% MeOH in DCM) to give the product as a white solid (115 mg, 68%).

^{1}H NMR ($CDCl_3$, 400 MHz) δ1.87 (s, 3H), 3.24 (m, 2H), 3.61 (s, 3H), 4.88 (td, *J* 5.4, 7.9, 1H,), 6.00 (s, 1H), 6.88 (s, 1H), 7.04 (t, *J* 7.4, 1H), 7.11 (t, *J* 7.5, 1H), 7.27 (d, *J* 8.1, 1H), 7.45 (d, *J* 7.9, 1H), 8.36 (s,1H); LCMS[a] Calc for $C_{14}H_{17}N_2O_3$ $(M+H)^+$ 261.1 found 261.2 R_t = 3.3 min

(*S*)-2-acetamido-3-(1H-indol-3-yl)-N-methylpropanamide (4)

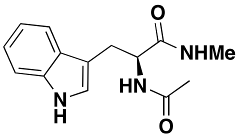

Acetic anhydride (75 μL, 0.79 mmol) was added dropwise to a solution of **2** (165 mg, 0.76 mmol) in pyridine (4 mL) and DCM (12 mL) at 4 °C. The reaction mixture was allowed to warm to room temperature and stirred for 4 hours and the solvent removed *in vacuo*. Water (50 mL) and DCM (50 mL) was added to residue and the aqueous layer was extracted with DCM (4 x 50 mL). The combined organic layers were washed with brine, dried over Na_2SO_4 and the solvent removed *in vacuo.* The crude was purified by silica chromatography (10% MeOH in DCM) to give the product as a white solid (146 mg, 74%).

^{1}H NMR ($CDCl_3$, 400 MHz) δ 1.99 (s, 3H), 2.66 (d, *J* 4.7, 3H), 3.16 (dd, *J* 8.2,14.4, 1H), 3.31 (dd, *J* 5.4, 14.4, 1H), 4.71 (dt, *J* 5.7, 7.9, 1H), 5.89 (s, 1H),6.49 (d, *J* 7.1, 1H), 7.05 (s, 1H), 7.14 (t, *J* 7.0, 1H), 7.21 (t, *J* 7.0, 1H), 7.38 (d, *J* 8.1, 1H), 7.69 (d, *J* 7.8, 1H), 8.31 (s, 1H); ^{13}C NMR (MeOD-d^4, 125 MHz) 22.5, 26.3, 29.1, 55.9, 111.1, 112.2, 119.3, 119.7, 122.4, 124.4, 128.8, 138.0, 173.1, 174.7; LCMS[a]: calcd for $C_{14}H_{18}N_3O_2$ $(M+H)^+$ 260.1, found 260.0, R_t = 2.8 min; HRMS: calcd for $C_{14}H_{18}N_3O_2$ $(M+H)^+$ 260.1399, found 260.1407;

General Procedure for preparation of N-Boc protected tryptophan esters (N-Boc-Try-OR)

N-Boc-Tryptophan (1eq) was dissolved in anhydrous dichloromethane. The substituted alcohol (1 eq) was added to the reaction followed by a catalytic amount of DMAP (5 mol%). The reaction was cooled to 0^oC and a solution of DCC (1.1 eq) in anhydrous dichloromethane was added dropwise over 10 mins. The reaction was allowed to warm to room temperature and a precipitate was observed after 20 mins. The reaction was stirred for a further 4-8 hours until no starting material was observed by TLC. The reaction was filtered to remove the DCC urea by-product and the solvent removed *in-vacuo*. The residue was placed onto a column of flash silica gel where the product was eluted with dichloromethane:ethyl acetate (1:0 to 9:1). The solvent was removed under reduced pressure to afford the product.

(*S*)-phenyl-2-((*tert*-butoxycarbonyl)amino)-3-(1H-indol-3-yl)propanoate

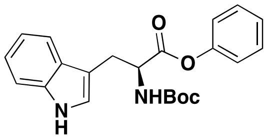

Off-White Solid, Yield: 84%, ^{1}H NMR ($CDCl_3$, 500 MHz): δ 1.45 (s, 9H), 3.44 (d, *J* 4.8, 2H), 4.89 (q, *J* 6.2, 1H), 5.17 (d, *J* 8.3, 1H), 6.92 (d, *J* 7.7, 2H), 7.10-7.15 (m, 2H), 7.19-7.24 (m, 2H), 7.33 (t, *J* 7.8, 2H), 7.38 (d, *J* 8.1, 1H), 7.65 (d, *J* 8.1, 1H), 8.15 (s, 1H) ^{13}C NMR ($CDCl_3$, 125 MHz): 28.0, 28.3, 54.5, 80.0, 110.1, 111.2, 115.3, 118.9, 121.3, 122.3, 122.9, 125.9, 127.7, 129.4, 129.6, 136.1, 150.4, 155.3, 170.9; υ_{max}/cm^{-1} (neat) 3399 (NH), 3336 (NH),1761, 1681 (C=O): LRMS[a]: calcd for $C_{22}H_{25}N_2O_4$ $(M+H)^+$ 381.1, found 381.1; HRMS (ESI): calcd for $C_{22}H_{25}N_2O_4$ $(M+H)^+$ 381.1815, found 381.1829.

(*S*)-benzyl-2-((*tert*-butoxycarbonyl)amino)-3-(1H-indol-3-yl)propanoate [3]

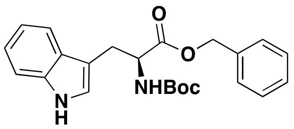

White solid, Yield: 58% 1H-NMR (400 MHz, $CDCl_3$) δ 1.34 (s, 9H), 3.20 (d, *J* 5.3, 2H), 4.61 (m, 1H), 5.00 (m, 2H), 6.71 (s, 1H), 7.02 (td, *J* 7.5,1.0, 1H), 7.10 (td, *J* 7.1, 1.1, 1H), 7.14 (m, 2H), 7.24 (m, 4H), 7.47 (d, *J* 7.9, 1H), 8.03 (s, 1H); ^{13}C-NMR (125 MHz, $CDCl_3$) δ 27.9, 28.3, 54.3, 67.0, 79.8, 110.1, 111.1, 118.8, 119.6, 122.2, 122.8, 127.7, 128.3, 128.4, 128.5, 135.3, 136.0, 155.2, 172.2; LCMS[a]: calcd for $C_{23}H_{27}N_2O_4$ $(M+H)^+$ 395.2, found 394.9, R_t = 4.6 min

(*S*)-phenethyl-2-((*tert*-butoxycarbonyl)amino)-3-(1H-indol-3-yl)propanoate

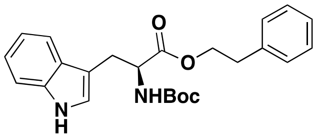

White solid, Yield: 79% ^{1}H NMR ($CDCl_3$, 400 MHz) δ 1.35 (s, 9H), 2.75 (t, *J* 7.0, 2H), 3.15 (m, 2H), 4.18 (m, 2H), 4.55 (dd, *J* 5.1,12.6, 1H), 6.70 (s, 1H), 7.00-7.25 (m, 9H), 7.41 (d, *J* 7.9, 1H), 8.01 (bs, 1H), ^{13}C NMR ($CDCl_3$, 125 MHz) δ 28.4, 28.8, 35.3, 54.7, 66.2, 80.2, 110.6, 111.6, 119.2, 120.0, 122.6, 123.1, 127.0, 128.1, 128.9, 129.4, 136.5, 138.0, 155.7, 172.6; LCMS[a]: calcd for $C_{24}H_{29}N_2O_4$ $(M+H)^+$ 409.2, found 409.6, R_t = 4.8 min; HRMS: calcd for $C_{24}H_{29}N_2O_4$ $(M+H)^+$ 409.2127, found 409.2135;

(*S*)-3-phenylpropyl 2-((*tert*-butoxycarbonyl)amino)-3-(1H-indol-3-yl)propanoate

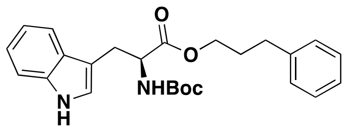

Off-White Solid, Yield: 82%, ^{1}H NMR ($CDCl_3$, 500 MHz) δ 1.44 (s, 9H), 1.79-1.93 (m, 2H), 2.55 (t, *J* 7.7, 2H), 3.29 (d, *J* 5.8, 2H), 4.02-4.12 (m, 2H), 4.66 (q, *J* 6.0, 1H), 5.09 (d, *J* 8.2, 1H), 6.99 (s, 2H), 7.07-7.16 (m, 2H), 7.17-7.22 (m, 2H), 7.26-7.29 (m, 2H), 7.33 (d, *J* 8.1, 1H), 7.58 (d, *J* 7.9, 1H), 8.15 (s, 1H); ^{13}C NMR ($CDCl_3$, 125 MHz) 28.2, 28.4, 29.9, 31.9, 54.3, 64.6, 79.8, 110.3, 111.2, 118.8, 119.6, 122.2, 122.7, 126.0, 127.7, 128.4, 128.5, 136.1, 141.0, 155.3, 172.4; υ_{max} /cm^{-1} (neat) 3397 (NH), 3317 (NH),1732, 1683 (C=O): LRMS[a]: calcd for $C_{25}H_{31}N_2O_4$ $(M+H)^+$ 423.2, found 423.4 HRMS (ESI): calcd for $C_{25}H_{31}N_2O_4$ $(M+H)^+$ 423.2285, found 423.2287.

(*S*)-*tert*-butyl (3-(1H-indol-3-yl)-1-oxo-1-(phenethylamino)propan-2-yl)carbamate

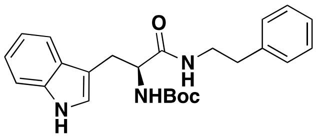

Clear oil, Yield: 68% ^{1}H-NMR ($CDCl_3$, 400 MHz) δ 1.33 (s, 9H), 2.44 (m, 2H) 3.04 (dd, *J* 7.8, 14.3, 1H), 3.19 (m, 2H), 3.31 (m, 1H), 4.29 (m, 1H), 5.12 (bs, 1H), 5.68 (bs, 1H), 6.82 (m, 2H), 6.87 (d, *J* 1.6, 1H),7.04 (t, *J* 7.9, 1H), 7.07-7.14 (m, 4H), 7.27 (d, *J* 8.1, 1H), 7.56 (d, *J* 7.8, 1H), 8.35 (bs, 1H); ^{13}C NMR ($CDCl_3$, 125 MHz) δ 28.7, 29.1, 35.8, 41.0, 55.8, 80.5, 111.0, 111.7, 119.3, 120.1, 122.6, 123.6, 126.8, 127.9, 128.0, 129.0, 136.7, 139.0, 155.9, 172.0; LCMS[b]: calcd for $C_{24}H_{30}N_3O_3$ $(M+H)^+$ 408.2, found 408.4, R_t = 2.3 min; HRMS: calcd for $C_{24}H_{30}N_3O_3$ $(M+H)^+$ 408.2287, found 408.2291

(*S*)-2-fluorophenethyl 2-((*tert*-butoxycarbonyl)amino)-3-(1H-indol-3-yl)propanoate

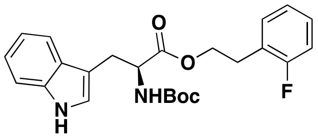

White Solid, Yield: 78%, ^{1}H NMR ($CDCl_3$, 400 MHz): δ 1.42 (s, 9H), 2.89 (t, *J* 6.9, 2H), 3.18-3.27 (m, 2H), 4.23-4.30 (m, 2H), 4.63 (br q, *J* 5.8, 1H), 5.05 (d, *J* 8.3, 1H), 6.85 (d, *J* 2.4, 1H), 7.00-7.05 (m, 2H), 7.10 (t, *J* 7.3, 2H), 7.16-7.23 (m 2H), 7.33 (d, *J* 8.2, 1H), 7.49 (d, *J* 7.9, 1H), 8.05 (s, 1H); ^{13}C NMR ($CDCl_3$, 100 MHz) δ 28.0, 54.3, 64.5, 79.8, 110.3, 111.1, 115.3 (d, *J* 21.9), 118.8, 119.6, 122.2, 122.6, 124.1, 124.5 (d, *J* 15.7), 127.7, 128.5 (d, *J* 8.0), 131.3, 136.1, 155.2, 161.2 (d, *J* 245.6), 172.1; ^{19}F NMR ($CDCl_3$, 376 MHz): δ -118.5 (Aryl F); υ_{max} /cm^{-1} (neat) 3297 (NH), 1727, 1685 (C=O): LRMS[a]: calcd for $C_{24}H_{28}N_2O_4F$ $(M+H)^+$ 427.2, found 427.4 HRMS (ESI): calcd for $C_{24}H_{28}N_2O_4F$ $(M+H)^+$ 427.2034, found 427.2031.

(*S*)-2-hydroxyphenethyl 2-((*tert*-butoxycarbonyl)amino)-3-(1H-indol-3-yl)propanoate

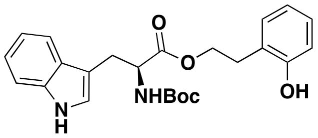

Clear oil, Yield: 65% ^{1}H-NMR (400 MHz, $CDCl_3$) δ 1.35 (s, 9H), 2.77 (m, 2H), 3.17 (m, 2H), 4.19 (m, 2H), 4.55 (m, 1H), 5.03 (m, 1H), 6.75 (m, 3H), 6.96 (dd, *J* 1.5, 7.5, 1H), 7.02 (t, *J* 7.8, 2H), 7.10 (td, *J* 1.1, 7.1, 1H), 7.24 (d, *J* 8.1, 1H), 7.43 (d, *J* 7.9, 1H), 8.04 (br s, 1H), ^{13}C-NMR (100 MHz, $CDCl_3$) δ 25.3, 28.7, 34.2, 54.8, 65.6, 83.0, 111.6, 111.7, 116.2, 119.2, 120.0, 120.9, 122.6, 122.7, 123.3, 123.6, 124.3, 128.6, 131.3, 146.9, 154.9, 173.0; IR (ATR): υ_{max} /cm^{-1} (neat) 1740 (C=O); LCMS[a]: calcd for $C_{24}H_{29}N_2O_5$ $(M+H)^+$ 425.2, found 425.2, R_t = 4.2 min; HRMS: calcd for $C_{24}H_{29}N_2O_5$ $(M+H)^+$ 425.2076, found 425.2079.

(*S*)-2-methylphenethyl 2-((tert-butoxycarbonyl)amino)-3-(1H-indol-3-yl)propanoate

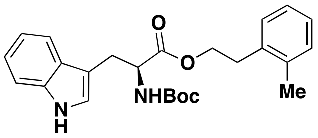

White Solid, Yield: 60%, ^{1}H NMR ($CDCl_3$, 400 MHz): δ 1.44 (s, 9H), 2.30 (s, 3H), 2.85 (t, *J* 7.3, 2H), 3.24 (t, *J* 4.9, 2H), 4.18-4.25 (m, 2H), 4.64 (q, *J* 5.7, 1H), 5.09 (d, *J* 8.3, 1H), 6.79 (d, *J* 2.1, 1H), 7.07-7.20 (m, 4H), 7.32 (d, *J* 8.1, 2H), 7.49 (d, *J* 7.9, 2H), 8.12 (s, 1H); ^{13}C NMR δ 19.4, 28.0, 32.1, 54.3, 65.0. 79.5, 110.1, 111.1, 118.7, 119.6, 122.2, 122.8, 126.1, 126.9, 129.7, 130.4, 135.7, 136.1, 136.4, 155.3, 172.1; υ_{max} /cm^{-1} (neat) 3297 (NH), 1729, 1687 (C=O); LRMS[a]: calcd for $C_{25}H_{30}NO_4Na$ $(M+Na)^+$ 445.2, found 445.1; HRMS (ESI): calcd for $C_{25}H_{31}NO_4$ $(M+H)^+$ 423.2285, found 423.2289.

(*S*)-3-methylphenethyl 2-((*tert*-butoxycarbonyl)amino)-3-(1H-indol-3-yl)propanoate

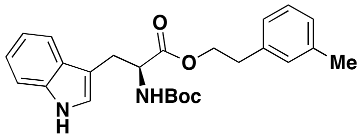

White Solid, Yield: 65%, ^{1}H NMR ($CDCl_3$, 400 MHz): δ 1.44 (s, 9H), 2.31 (s, 3H), 2.80 (t, *J* 7.0, 2H), 3.24 (t, *J* 6.2, 2H), 4.22-4.27 (m, 2H), 4.64 (q, *J* 5.7, 1H), 5.08 (d, *J* 8.3, 1H), 6.79 (s, 1H), 6.93-7.23 (m, 6H), 7.32 (d, *J* 8.1, 1H), 7.50 (d, *J* 7.8, 1H), 8.15 (s, 1H). ^{13}C NMR ($CDCl_3$, 100 MHz): δ 21.4, 27.9, 28.4, 34.8, 54.4, 65.9, 79.8, 110.2, 111.2, 118.8, 119.6, 122.2, 122.7, 125.9, 127.4, 127.7, 129.8, 136.0, 137.5, 138.2, 155.3, 172.2. υ_{max}/cm^{-1} (neat) 3297 (NH) 1733, 1682 (C=O): LRMS[a]: calcd for $C_{25}H_{30}NO_4Na$ $(M+Na)^+$ 445.2, found 445.2; HRMS (ESI): calcd for $C_{25}H_{31}NO_4$ $(M+H)^+$ 423.2285, found 423.2317.

(*S*)-4-methylphenethyl 2-((*tert*-butoxycarbonyl)amino)-3-(1H-indol-3-yl)propanoate

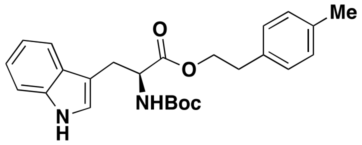

Off-White Solid, Yield: 38%, ^{1}H NMR ($CDCl_3$, 400 MHz): δ 1.42 (s, 9H), 2.30 (s, 3H), 2.79 (t, *J* 7.0, 2H), 3.23 (t, *J* 5.8, 2H), 4.19-4.25 (m, 2H), 4.62 (q, *J* 5.7, 1H), 5.05 (d, *J* 8.3, 1H), 6.80 (s, 1H), 7.02-7.20 (m, 6H), 7.33 (d, *J* 8.1, 1H), 7.49 (d, *J* 7.9, 1H), 8.04 (s, 1H); ^{13}C NMR ($CDCl_3$, 100 MHz): δ 21.0, 28.1, 28.4, 34.4, 54.3, 66.0, 79.8, 110.2, 111.2, 118.1, 119.6, 122.2, 122.7, 127.8, 128.8, 129.2, 134.5, 136.0, 136.2, 155.2, 172.2; υ_{max} /cm^{-1} (neat) 3297 (NH), 1736, 1685 (C=O): LRMS[a]: calcd for $C_{25}H_{30}NO_4Na$ $(M+Na)^+$ 445.2, found 445.0; HRMS (ESI): calcd for $C_{25}H_{31}NO_4$ $(M+H)^+$ 423.2285, found 423.2286.

(S)-4-methoxyphenethyl 2-((tert-butoxycarbonyl)amino)-3-(1H-indol-3-yl)propanoate

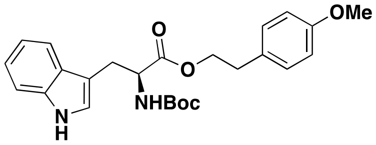

Off-White Solid, Yield: 68%, ^{1}H NMR ($CDCl_3$, 400 MHz): δ 1.42 (s, 9H), 2.76 (t, *J* 7.0, 2H), 3.23 (t, *J* 5.2, 2H), 3.76 (s, 3H), 4.18-4.22 (m, 2H), 4.62 (q, *J* 5.7, 1H), 5.05 (d, *J* 8.3, 1H), 6.81 (s, 1H), 7.01, 7.23 (m, 6H), 7.33 (d, *J* 8.3, 1H), 7.50 (d, *J* 7.8, 1H), 8.03 (s, 1H); ^{13}C NMR ($CDCl_3$, 100 MHz): δ 28.0, 28.4, 34.0, 54.2, 55.2, 66.0, 79.8, 110.2, 111.1, 113.9, 118.8, 119.6, 122.2, 122.7, 127.8, 129.6, 129.9, 136.0, 155.2, 158.4, 172.2; υ_{max}/cm^{-1} (neat) 3297 (NH), 1731, 1686 (C=O): LRMS[a]: calcd for $C_{25}H_{30}N_2O_5Na$ $(M+Na)^+$ 461.2, found 461.1; HRMS (ESI): calcd for $C_{25}H_{31}N_2O_5$ $(M+H)^+$ 439.2234, found 439.2249.

(*S*)-4-bromophenethyl 2-((*tert*-butoxycarbonyl)amino)-3-(1H-indol-3-yl)propanoate

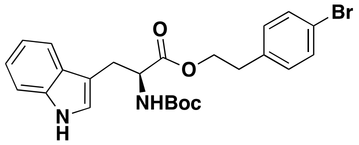

Off-White Solid, Yield: 46%, ^{1}H NMR ($CDCl_3$, 400 MHz) δ 1.42 (s, 9H), 2.73 (t, *J* 7.1, 2H), 3.21 (d, *J* 4.7, 2H), 4.16-4.22 (m, 2H), 4.60 (q, *J* 6.5, 1H), 5.04 (d, *J* 8.4, 1H), 6.82 (s, 1H), 6.94 (d, *J* 6.9, 2H), 7.07-7.20 (m, 2H), 7.31-7.36 (m, 3H), 7.49 (d, *J* 8.0, 1H), 8.14 (s, 1H); ^{13}C NMR ($CDCl_3$, 100 MHz) δ 28.0, 28.4, 34.3, 54.3, 60.5, 65.4, 79.9, 110.1, 111.3, 118.7, 119.6, 120.5, 122.2, 122.7, 127.6, 130.6, 131.6, 136.0, 136.6, 155.2, 172.3: υ_{max} /cm^{-1} (neat) 3300 (NH), 1731, 1690 (C=O): LRMS: calcd for $C_{24}H_{28}N_2O_4Br$ $(M+Na)^+$ 511.4, found 511.1; HRMS (ESI): calcd for $C_{24}H_{28}N_2O_4Br$ $(M+H)^+$ 487.1233, found 487.1236.

(*S*)-2-cyclohexylethyl 2-((tert-butoxycarbonyl)amino)-3-(1H-indol-3-yl)propanoate

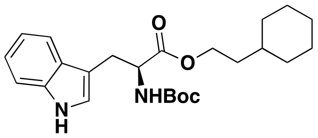

Gum, Yield: 82%, ^{1}H NMR ($CDCl_3$, 500 MHz): δ 0.83-0.90 (m, 2H), 1.08-1.45 (m, 13H, 1.63-1.68 (m, 6H), 3.24-3.32 (m, 2H), 4.02-4.12 (m, 2H), 4.63 (dd, *J* 7.6, 5.8, 1H), 5.08 (d, *J* 7.8, 1H), 6.99 (s, 1H), 7.11 (t, *J* 7.4, 1H), 7.18 (t, *J* 8.2, 1H), 7.25 (s, 1H), 7.34 (d, *J* 8.0, 1H), 7.55 (d, *J* 8.0, 1H), 8.1 (s, 1H); ^{13}C NMR ($CDCl_3$, 125 MHz): 26.1, 26.4, 28.0, 28.3, 33.0, 34.4, 35.7, 54.2, 63.6, 79.7, 110.3, 111.1, 118.8, 119.6, 122.2, 122.7, 127.7, 136.1, 155.3, 172.4. LRMS[b]: calcd for $C_{24}H_{35}N_2O_4$ $(M-H)^+$ 415.2, found 415.5; HRMS (ESI): calcd for $C_{24}H_{35}N_2O_4$ $(M+H)^+$ 415.2597, found 415.2620.

(*S*)-2-(1H-indol-3-yl)ethyl 2-((tert-butoxycarbonyl)amino)-3-(1H-indol-3-yl)propanoate

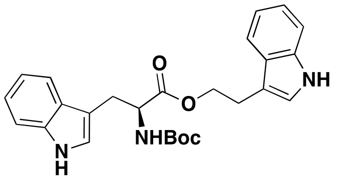

Pale yellow oil, Yield: 72% ^{1}H-NMR ($CDCl_3$, 400 MHz) 1.47 (s, 9H). 3.03 (t, *J* 6.8, 2H), 3.25 (m, 2H), 4.35 (t, *J* 6.8, 2H), 4.69 (m, 1H), 5.14 (d, *J* 7.9, 1H), 6.72 (s, 1H), 6.88 (d, *J* 2.1, 1H), 7.09 (t, *J* 7.4, 1H), 7.25-7.15 (m, 3H), 7.32 (d, *J* 8.1, 1H), 7.36 (d, *J* 8.1, 1H), 7.52 (d, *J* 7.9, 1H), 7.59 (d, *J* 7.8, 1H), 8.05 (bs, 1H), 8.13 (bs, 1H); ^{13}C NMR ($CDCl_3$, 100 MHz) 24.9, 28.4, 28.7, 55.6, 68.8, 80.2, 110.4, 111.6, 112.0, 119.1, 119.2, 119.8, 119.9, 122.5, 122.8, 123.3, 127.7, 128.1, 136.5, 136.6, 155.7, 172.8; LCMS[b]: calcd for $C_{26}H_{30}N_3O_4$ $(M+H)^+$ 448.2, found 448.4, R_t = 2.45 min; HRMS: calcd for $C_{26}H_{30}N_3O_4$ $(M+H)^+$;448.2236, found 448.2245.

General Procedure for preparation of tryptophan esters (Try-OR)

The N-Boc tryptophan ester (1 eq.) was dissolved in anhydrous diethyl ether. Hydrogen chloride gas (**CAUTION: Highly corrosive and all handling should be done in a fumehood**) was bubbled through the solution for 10 mins and precipitation of the product was observed. The reaction was stirred for 1 hour under an atmosphere of hydrogen chloride. The reaction was flushed with nitrogen gas for 5 minutes and the product was collected by filtration and was used without further purification.

(*S*)-phenyl 2-amino-3-(1*H*-indol-3-yl)propanoate hydrochloride (5a)

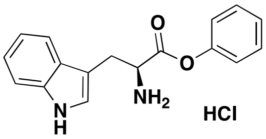

White Solid, Yield: 76%, ^{1}H NMR (DMSO-d^{6}, 500 MHz): δ 3.36-3.55 (m, 2H) (overlap with the H_2O signal in DMSO-d^{6}), 4.46 (dd, *J* 7.8, 5.8, 1H), 6.90 (dd, *J* 7.7, 1.5, 2H), 7.02 (t, *J* 7.0, 1H), 7.12 (t, *J* 7.6, 1H), 7.21-7.28 (m, 1H), 7.32-7.47 (m, 4H), 7.64 (d, *J* 7.9, 1H), 8.84 (s, 3H, protonated NH_2), 11.15 (s, 1H); ^{13}C NMR (DMSO-d^{6}, 125 MHz): 26.9, 53.5, 107.0, 112.1, 118.7, 119.1, 121.7, 125.5, 126.8, 127.4, 130.0, 136.7, 150.0, 168.7; υ_{max} /cm^{-1} (neat) 1750 (C=O); LRMS[a]: calcd for $C_{17}H_{17}N_2O_2$ $(M+H)^+$ 281.1 found 281.2; HRMS (ESI): calcd for $C_{17}H_{17}N_2O_2$ $(M+H)^+$ 281.1291 found 281.1299.

(*S*)-benzyl-2-amino-3-(1H-indol-3-yl)propanoate hydrochloride (5b)

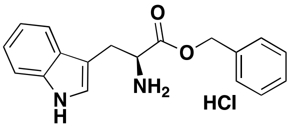

White solid, Yield: 91% ^{1}H NMR (DMSO-d^{6}, 400 MHz) δ 3.31 (m, 2H). 4.30 (t, *J* 6.5 1H), 5.06 (d, *J* 12.4, 1H), 5.15 (d, *J* 12.4, 1H), 7.01 (t, *J* 7.1, 1H), 7.11 (t, *J* 7.1, 1H), 7.34 (m, 3H), 7.21 (m, 3H), 7.39 (d, *J* 8.1, 1H), 7.52 (d, *J* 7.9, 1H), 8.54 (bs, 3H), 11.08 (s, 1H); ^{13}C NMR (DMSO-d^{6}, 125 MHz) δ 26.4, 52.8, 67.1, 106.5, 111.7, 118.1, 118.8, 121.3, 124.9, 125.0, 127.0, 128.2, 128.5, 135.0, 136.3, 169.4; LCMS[a]: calcd for $C_{18}H_{19}N_2O_2$ $(M+H)^+$ 295.1, found 295.0, R_t = 3.2 min; HRMS: calcd for $C_{18}H_{19}N_2O_2$ $(M+H)^+$ 295.1447, found 295.1443;

(*S*)-phenethyl 2-amino-3-(1H-indol-3-yl)propanoate hydrochloride (5c)

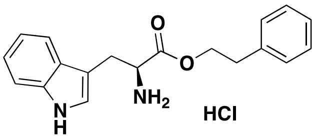

White solid, Yield: 74% ^{1}H NMR (DMSO-d^{6}, 400 MHz) δ 2.75 (m, 2H). 3.24 (m, 2H), 4.17 (t, *J* 6.4, 1H), 4.22 (t, *J* 6.8, 2H), 7.00 (t, *J* 7.0, 1H), 7.10 (t, *J* 7.1, 1H), 7.16 (m, 3H), 7.22 (t, *J* 7.2, 1H), 7.28 (t, *J* 7.2, 2H), 7.38 (d, *J* 8.1, 1H), 7.41 (d, *J* 7.9, 1H), 8.52 (bs, 3H); ^{13}C NMR (DMSO-d^{6}, 100 MHz) δ 26.3, 34.0, 52.8, 66.2, 106.5, 111.6, 118.0, 118.7, 121.3, 124.9.

126.6, 127.0, 128.5, 129.0, 136.3, 137.6, 169.5; LCMS[a]: calcd for $C_{19}H_{21}N_2O_2$ $(M+H)^+$ 309.2, found 309.5, R_t = 3.5 min; HRMS: calcd for $C_{19}H_{21}N_2O_2$ $(M+H)^+$ 309.1603, found 309.1603.

(*S*)-3-phenylpropyl 2-amino-3-(1H-indol-3-yl)propanoate hydrochloride (5d)

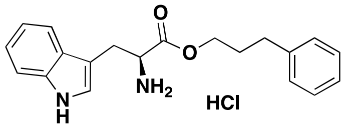

White Solid, Yield: 75%, ^{1}H NMR (DMSO-d^6, 500 MHz): δ 1.70-1.77 (m, 2H), 2.41-2.48 (m, 2H, some overlap with DMSO peak), 3.23-3.28 (m, 2H, some overlap with the H_2O), 3.94-4.06 (m, 2H), 4.23 (t, *J* 6.6, 1H), 6.99 (t, *J* 7.5, 1H), 7.07-7.11 (m, 3H), 7.15-7.19 (m, 1H), 7.22-7.31 (m, 3H), 7.36 (d, *J* 8.1, 1H), 7.52 (d, *J* 7.8, 1H), 8.55 (br s, 2H), 11.10 (s, 1H); ^{13}C NMR (DMSO-d^6, 125 MHz): δ 26.8, 29.8, 31.4, 53.2, 65.3, 107.0, 112.0, 118.5, 119.0, 121.6, 125.3, 126.3, 127.4, 128.7, 128.8, 136.7, 141.1, 169.9; υ_{max}/cm^{-1} (neat) 1742 (C=O); LRMS: calcd for $C_{20}H_{23}N_2O_2$ $(M+H)^+$ 323.2 found 323.5 HRMS (ESI): calcd for $C_{20}H_{23}N_2O_2$ $(M+H)^+$ 323.1697, found 323.1737.

(*S*)-2-amino-3-(1H-indol-3-yl)-N-phenethylpropanamide hydrochloride (5e)

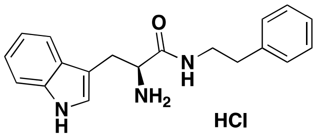

White solid, Yield: 98% ^{1}H NMR (DMSO-d^6, 400 MHz) δ 2.62 (t, *J* 7.4, 2H). 3.20 (m, 3H), 3.37 (m, 1H), 3.96 (m, 1H), 7.00 (t, *J* 7.1, 1H), 7.09 (t, *J* 7.1, 1H), 7.21 (m, 6H), 7.38 (d, *J* 8.1, 1H), 7.68 (d, *J* 7.9, 1H), 8.35 (br s, *J* 2.9, 3H), 8.78 (t, *J* 5.5, 1H), 11.13 (d, *J* 1.4, 1H); ^{13}C NMR (DMSO-d^6, 100 MHz) δ 27.6, 35.1, 40.8, 53.2, 107.1, 111.8, 118.7, 119.0, 121.4, 125.1, 126.5, 127.5, 128.6, 128.9, 136.6, 139.5, 168.6; IR (ATR): υ_{max} /cm^{-1} (neat) 1638 (C=O); LCMS[b]: calcd for $C_{19}H_{22}N_3O$ $(M+H)^+$ 308.2, found 308.3, R_t = 1.4 min; HRMS: calcd for $C_{19}H_{22}N_3O$ $(M+H)^+$ 308.1763, 308.1754;

(*S*)-2-fluorophenethyl 2-amino-3-(1*H*-indol-3-yl)propanoate hydrochloride (5f)

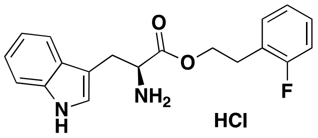

Off-White Solid, Yield: 56%, ^{1}H NMR (DMSO-d^6, 400 MHz): δ 2.76-2.91 (m, 2H), 3.20-3.30 (m, 2H), 4.14 (t, *J* 6.3, 1H), 4.19-4.25 (m, 2H), 6.98 (td, *J* 7.3, 1.1, 1H), 7.06-7.31 (m, 6H), 7.38 (t, *J* 7.5, 2H), 8.64 (br s, 3H), 11.13 (s, 1H); ^{13}C NMR (DMSO-d^6, 100 MHz): δ 26.5, 27.8, 53.1, 65.1, 106.7, 112.0, 115.6 (d, *J* 21.7), 118.3, 119.0, 121.6, 124.5 (d, *J* 15.6), 124.9 (d, *J* 3.3), 125.4, 127.3, 129.2 (d, *J* 8.1), 132.0 (d, *J* 4.5), 136.6, 161.1 (d, *J* 243.7), 169.7; ^{19}F NMR (DMSO-d^6, 376 MHz): δ -118.3 (Aryl F); υ_{max} /cm^{-1} (neat) 1743 (C=O): LRMS[a]: calcd for

$C_{19}H_{20}FN_2O_2$ $(M+H)^+$ 327.1, found 327.0; HRMS (ESI): calcd for $C_{19}H_{20}FN_2O_2$ $(M+H)^+$ 327.1510, found 327.1516.

(*S*)-2-hydroxyphenethyl 2-amino-3-(1H-indol-3-yl)propanoate hydrochloride (5g)

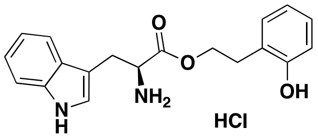

White solid, Yield: 72% ^{1}H-NMR (DMSO-d^6, 400 MHz) 2.77 (m, 2H), 3.25 (m, 2H), 4.19 (bs, 1H), 4.22 (t, *J* 7.0, 2H), 6.70 (dt, *J* 7.4, 1.0, 1H), 6.85 (dd, *J* 8.0 0.8, 1H), 7.02 (m, 3H), 7.10 (td, *J* 7.5, 0.9, 1H), 7.18 (d, *J* 2.3, 1H), 7.38 (d, *J* 8.0, 1H), 7.43 (d, *J* 7.8, 1H), 8.50 (bs, 3H), 9.54 (s, 1H), 11.08 (s, 1H); ^{13}C-NMR (DMSO-d^6,125 MHz) 25.4, 29.1, 52.8, 64.8, 106.4, 111.6, 115.0, 118.3, 118.7, 118.9, 121.2, 123.2, 125.0, 126.9, 127.8, 130.7, 136.2, 155.5, 169.3; LCMS[a]: calcd for $C_{19}H_{21}N_2O_3$ $(M+H)^+$ 325.2, found 325.0, R_t = 3.2 min; HRMS: calcd for $C_{19}H_{21}N_2O_3$ $(M+H)^+$ 325.1552, found 325.1560;

(*S*)-2-methylphenethyl 2-amino-3-(1H-indol-3-yl)propanoate hydrochloride (5h)

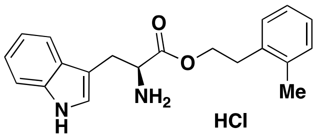

Off-White Solid, Yield: 84%, ^{1}H NMR (DMSO-d^6, 400 MHz): δ 2.24 (s, 3H), 2.69-2.81 (m, 2H), 3.22-3.31 (m, 2H, some overlap with the H_2O peak), 4.14-4.20 (m, 3H), 6.98 (t, *J* 7.5, 1H), 7.05-7.18 (m, 6H), 7.38 (t, *J* 8.6, 2H), 8.57 (br s, 3H), 11.10 (s, 1H); ^{13}C NMR (DMSO-d^6, 100 MHz): δ 19.4, 26.2, 31.6, 53.3, 65.5, 106.8, 112.0, 118.4, 119.0, 121.6, 125.4, 126.4, 127.1, 127.4, 129.9, 130.6, 135.9, 136.5, 136.6, 169.9; υ_{max} /cm^{-1} (neat) 1743 (C=O); LRMS[a]: calcd for $C_{20}H_{22}N_2O_2Na$ $(M+Na)^+$ 345.2, found 345.0; HRMS (ESI): calcd for $C_{20}H_{23}N_2O_2$ $(M+H)^+$ 323.1760, found 323.1756.

(*S*)-3-methylphenethyl 2-amino-3-(1H-indol-3-yl)propanoate hydrochloride (5i)

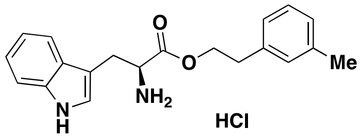

Off-White Solid, Yield: 60%, ^{1}H NMR (DMSO-d^6, 400 MHz): δ 2.24 (s, 3H), 2.61-2.75 (m, 2H), 3.13-3.21 (m, 2H), 4.15-4.23 (m, 3H), 6.93-7.01 (m, 4H), 7.06-7.16 (m, 3H), 7.37 (dd, *J* 12.2, 8.0, 2H), 8.23 (br s, 3H), 11.03 (br s, 1H); ^{13}C NMR (DMSO-d^6, 100 MHz): 21.4, 26.8, 34.3, 53.2, 66.6, 106.9, 112.0, 119.1, 121.7, 125.3, 126.4, 127.6, 128.7, 130.0, 136.6, 137.8, 137.9, 170.0. IR (ATR): υ_{max} /cm^{-1} (neat) 1741 (C=O): LRMS[a]: calcd for $C_{20}H_{22}N_2O_2Na$ $(M+Na)^+$ 345.2, found 345.2; HRMS (ESI): calcd for $C_{20}H_{23}N_2O_2$ $(M+H)^+$ 323.1760, found 323.1743.

(*S*)-4-methylphenethyl 2-amino-3-(1H-indol-3-yl)propanoate hydrochloride (5j)

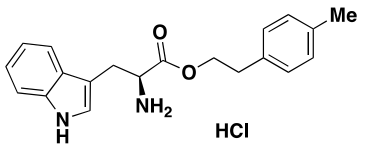

Off-White Solid, Yield: 79%, ^{1}H NMR (DMSO-d^6, 400 MHz): δ 2.23 (s, 3H), 2.61-2.74 (m, 2H), 3.13-3.21 (m, 2H), 4.15-4.20 (m, 3H), 6.97-7.12 (m, 7H), 7.37 (dd, *J* 10.7, 8.1, 2H), 8.14 (br s, 3H), 11.02 (br s, 1H); ^{13}C NMR (DMSO-d^6, 100 MHz): 21.1, 26.9, 33.9, 53.2, 66.6, 106.9, 112.0, 118.4, 119.1, 121.7, 125.2, 127.4, 129.2, 129.4, 134.8, 135.9, 136.6, 170.0; υ_{max}/cm^{-1} (neat) 1744 (C=O): LRMS[a]: calcd for $C_{20}H_{22}N_2O_2Na$ $(M+Na)^+$ 345.2, found 345.1; HRMS (ESI): calcd for $C_{20}H_{23}N_2O_2$ $(M+H)^+$ 323.1760, found 323.1794.

(*S*)-4-methoxyphenethyl 2-amino-3-(1H-indol-3-yl)propanoate hydrochloride (5k)

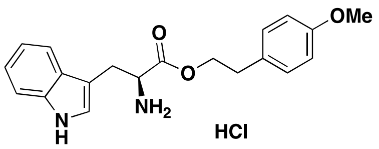

White Solid, Yield: 68%, ^{1}H NMR (DMSO-d^6, 500 MHz): δ 2.60-2.71 (m, 2H), 2.17-2.36 (m, 2H), 3.68 (s, 3H), 4.15 (t, *J* 6.9, 3H), 6.80 (d, *J* 8.6, 2H), 6.98 (t, *J* 7.5, 1H), 7.02-7.11 (m, 3H), 7.15 (d, *J* 2.4, 1H), 7.37 (dd, *J* 16.9, 8.0, 2H), 8.45 (br s,3H), 11.06 (s, 1H); ^{13}C NMR (DMSO-d^6, 125 MHz): 26.7, 33.5, 53.2, 55.5, 66.8, 106.9, 112.0, 114.2, 118.4, 119.0, 121.6, 125.3, 127.4, 129.8, 130.3, 136.6, 158.3, 169.8; υ_{max} /cm^{-1} (neat) 1743 (C=O): LRMS[a]: calcd for $C_{20}H_{23}N_2O_3$ $(M+H)^+$ 339.2, found 339.4; HRMS (ESI): calcd for $C_{20}H_{23}N_2O_3$ $(M+H)^+$ 339.1709, found 339.1708.

(*S*)-4-bromophenethyl 2-amino-3-(1H-indol-3-yl)propanoate hydrochloride (5l)

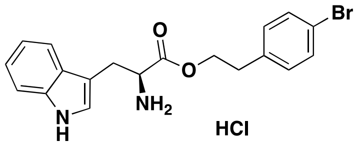

White Solid, Yield: 67%, ^{1}H NMR (DMSO-d^6, 400 MHz): δ 2.61-2.76 (m, 2H), 3.21 (qd, *J* 14.8, 6.4, 2H), 4.13-4.19 (m, 3H), 6.98 (t, *J* 7.5, 1H), 7.06-7.09 (m, 3H), 7.16 (d, *J* 2.4, 1H), 7.35-7.43 (m, 4H), 8.47 (br s, 3H), 11.07 (br s, 1H); ^{13}C NMR (DMSO-d^6, 100 MHz): 26.7, 33.7, 53.1, 66.2, 106.9, 112.0, 118.4, 119.1, 120.0, 121.7, 125.3, 127.4, 131.7, 136.6, 137.6, 169.8 υ_{max} /cm^{-1} (neat) 1745 (C=O): LRMS[a]: calcd for $C_{19}H_{20}N_2O_2Br$ $(M+H)^+$ 387.1, found 387.4 HRMS (ESI): calcd for $C_{19}H_{20}N_2O_2Br$ $(M+H)^+$ 387.0709, found 387.0707.

(*S*)-2-cyclohexylethyl 2-amino-3-(1H-indol-3-yl)propanoate hydrochloride (5m)

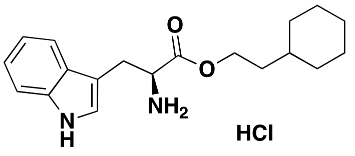

Off-white solid, Yield: 72%, ^{1}H NMR (DMSO-d^6, 500 MHz): δ 0.77-0.79 (m, 2H), 1.05-1.15 (m 4H), 1.28 (q, J 6.7, 2H), 1.52-1.48 (m, 4H), 3.99-4.05 (m, 2H), 4.20 (t, J 6.6, 1H), 6.98 (t, J 7.6, 1H), 7.07 (t, J 7.3, 1H), 7.20 (s, 1H), 7.34 (d, J 8.0, 1H), 7.46 (d, J, 7.8, 1H), 8.42(br s, 3H), 11.04 (s, 1H); ^{13}C NMR (DMSO-d^6, 125 MHz) δ 25.6, 26.0, 26.4, 32.2, 32.5, 35.1, 52.8, 63.7, 106.5, 111.7, 117.9, 118.7, 121.3, 124.8, 127.9, 136.3, 169.6; IR (ATR): υ_{max} /cm^{-1} (neat) 1740 (C=O); LRMS[a]: rt = 3.0 mins, calcd for $C_{19}H_{27}N_2O_2$ (M+H)$^+$ 315.2, found 315.4; HRMS (ESI): calcd for $C_{19}H_{27}N_2O_2$ (M+H)$^+$ 315.2073, found 315.2091.

Note: In the ^{1}H NMR one of the aliphatic signals appears between 3.25-3.40 ppm. However this overlaps with the H_2O signal from the DMSO-d^6. Confirmation of this is seen with the interaction with the signal at 4.20 ppm using the COSY spectrum.

(*S*)-2-(1H-indol-3-yl)ethyl 2-amino-3-(1H-indol-3-yl)propanoate hydrochloride (5n)

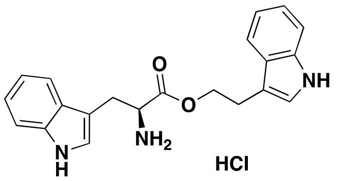

White solid, Yield: 75% ^{1}H-NMR (DMSO-d^6,400 MHz) δ 2.91 (m, 2H), 3.24 (m, 2H), 4.26 (m, 3H), 6.94 (td, *J* 7.5, 0.8, 1H,) 6.97 (td, *J* 7.4, 0.9, 1H), 7.08 (m, 2H), 7.15 (d, *J* 2.3, 1H), 7.20 (d, *J* 2.3, 1H), 7.37 (m, 3H), 7.49 (d, *J* 7.8,1H), 8.39 (bs, 3H), 10.89 (s, 1H), 11.05 (s, 1H), ^{13}C-NMR (DMSO-d^6, 125 MHz) δ 24.0, 26.3, 55.0, 66.0, 106.3, 109.6, 111.5, 111.6, 118.0, 118.2, 118.5, 118.7, 121.1, 121.3, 123.4, 125.0, 126.7, 127.1, 136.2, 136.3, 169.6; LCMS[a]: calcd for $C_{21}H_{22}N_3O_2$ (M+H)$^+$ 348.2, found 348.0, R_t = 3.4 min; HRMS: calcd for $C_{21}H_{22}N_3O_2$ (M+H)$^+$ 348.1712, found 348.1722.

General Procedure for preparation of N-substituted tryptophan esters

The tryptophan ester (1 eq.) was dissolved in anhydrous diethyl ether. Triethylamine (2 eq) was added to the reaction. This was followed by the corresponding carbonyl chloride (1 eq) which was added in dropwise over 10 mins. The reaction was stirred under an atmosphere of nitrogen until it was deemed complete by TLC (4-12 h). The reaction was dissolved with dichloromethane and washed with brine. The organic layer was separated and dried over sodium sulphate. The solvent was removed under reduced pressure and the residue placed onto a column of flash silica gel. The product was eluted with a gradient solvent mixture of dichloromethane: ethyl acetate 1:0 to 8:2. The solvent was removed under reduced *in-vacuo* to afford the title compound.

(*S*)-phenethyl 2-benzamido-3-(1H-indol-3-yl)propanoate (6a)

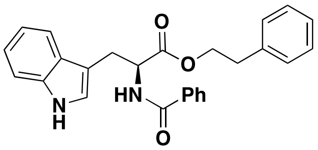

Clear oil, Yield: 84% ^{1}H NMR ($CDCl_3$, 400 MHz) δ 2.77 (m, 2H). 3.31 (m, 2H) 4.21 (m, 2H), 5.06 (m, 1H), 6.69 (d, *J* 7.7 1H), 6.74 (d, *J* 2.3, 1H), 7.06 (t, *J* 7.1, 1H), 7.37-7.11 (m, 9H), 7.47 (m, 2H), 7.65 (dd, *J* 1.2, 8.3, 2H), 8.37 (bs, 1H); ^{13}C-NMR ($CDCl_3$, 100 MHz) δ 27.7, 34.9, 53.7, 66.0, 109.8, 111.4, 118.7, 119.7, 122.2, 123.0, 126.7, 127.1, 127.7, 128.7, 128.6, 129.0, 131.7, 133.8, 136.2, 137.6, 167.0, 171.8; LCMS[a]: calcd for $C_{26}H_{25}N_2O_3$ $(M+H)^+$ 413.2, found 413.6, R_t = 4.6 min; HRMS: calcd for $C_{26}H_{25}N_2O_3$ $(M+H)^+$ 413.1860, found 413.1887.

(*S*)-2-fluorophenethyl acetyl-tryptophanate (6b)

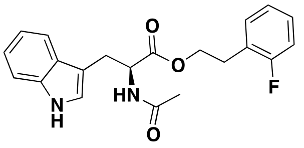

White Solid, Yield: 90%, ^{1}H NMR ($CDCl_3$, 400 MHz): δ 1.92 (s, 3H), 2.91 (t, *J* 6.7, 2H), 3.25 (qd, *J* 14.8, 5.2, 2H), 4.21-4.32 (m, 2H), 4.90-4.94 (m, 1H), 6.01 (d, *J* 7.8, 1H), 6.76 (s, 1H), 6.99-7.23 (m, 6H), 7.33 (d, *J* 8.1, 1H), 7.45 (d, *J* 8.1, 1H), 8.22 (br s, 1H); ^{13}C NMR ($CDCl_3$, 100 MHz): δ 23.2, 27.6, 28.4, 53.2, 64.7, 109.9, 111.3, 115.4 (d, *J* 21.9), 118.5, 119.7, 122.2, 122.7, 124.4 (d, *J* 3.5), 124.4 (d, *J* 15.7), 127.7, 128.6 (d, *J* 8.1), 131.3 (d, *J* 4.6), 136.0, 161.2 (d, *J* 245.4), 169.8, 171.8; ^{19}F NMR ($CDCl_3$, 376 MHz): δ -118.5 (Aryl F); υ_{max} /cm^{-1} (neat) 1727, 1656 (C=O): LRMS[a]: calcd for $C_{21}H_{21}N_2O_3FNa$ $(M+Na)^+$ 391.2, found 391.0; HRMS (ESI): calcd for $C_{21}H_{22}N_2O_3F$ $(M+H)^+$ 369.1615, found 369.1624.

(*S*)-phenethyl 3-(1H-indol-3-yl)-2-(3-nitrobenzamido)propanoate (6c)

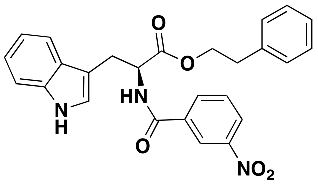

Off-White Solid, Yield: 71%, ^{1}H NMR ($CDCl_3$, 400 MHz): δ 2.86-2.93 (m, 2H), 3.41 (qd, *J* 14.8, 5.2, 2H), 4.30-4.40 (m, 2H), 5.13 (dt, *J* 7.8, 5.1, 1H), 6.67 (d, *J* 7.8, 1H), 6.72 (d, *J* 2.3, 1H), 7.07 (t, *J* 7.5, 1H), 7.15-7.36 (m, 8H), 7.47 (d, *J* 7.8, 1H), 7.54 (d, *J* 8.0, 1H), 7.92 (dt, *J* 7.8, 1.3, 1H), 8.09 (s, 1H), 8.31 (ddd, *J* 8.2, 2.3, 1.0, 1H), 8.44 (t, *J* 2.0, 1H); ^{13}C NMR ($CDCl_3$, 100 MHz): δ 27.4, 34.9, 53.9, 66.2, 109.7, 111.4, 118.4, 119.9, 122.1, 122.5, 122.8, 126.2, 126.5, 126.7, 127.6, 128.6, 129.0, 129.7, 133.0, 135.5, 136.0, 137.5, 138.5, 148.2, 164.5, 171.4; υ_{max} /cm^{-1} (neat) 1727, 1656 (C=O): LRMS[a]: calcd for $C_{26}H_{24}N_2O_5$ $(M+H)^+$ 458.1, found 458.0; HRMS (ESI): calcd for $C_{26}H_{24}N_2O_5$ $(M+H)^+$ 458.1717, found 458.1703.

(*S*)-phenethyl 2-(cyclohexanecarboxamido)-3-(1H-indol-3-yl)propanoate (6d)

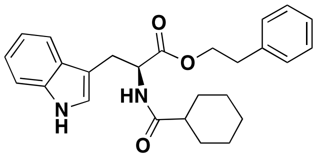

Off-White Solid, Yield: 76%, ^{1}H NMR ($CDCl_3$, 400 MHz): δ 1.14-1.25 (m, 3H) , 1.32-1.38 (m, 2H), 1.61-1.79 (m, 4H), 2.01 (tt, *J* 11.8, 3.4, 1H), 2.83-2.86 (m, 2H), 3.26 (d, *J* 5.6, 2H), 4.21-4.32 (m, 2H), 4.92-4.96 (m, 2H), 5.95 (d, *J* 7.9, 1H), 6.73 (s, 1H), 7.05-7.31 (m, 7H), 7.34 (d, *J* 8.1, 1H), 7.47 (d, *J* 7.9, 1H), 8.15 (br s, 1H); ^{13}C NMR ($CDCl_3$, 100 MHz): δ 25.6, 25.7, 27.6, 29.4, 29.5, 34.8, 45.2, 52.8, 65.9, 110.1, 111.2, 118.7, 119.5, 122.2, 122.7, 126.6, 127.7, 128.5, 128.9, 136.0, 137.6, 172.0, 175.6; υ_{max} /cm^{-1} (neat) 1726, 1645 (C=O): LRMS[a]: calcd for $C_{26}H_{31}N_2O_3$ $(M+H)^+$ 419.2, found 419.0; HRMS (ESI): calcd for $C_{26}H_{31}N_2O_3$ $(M+H)^+$ 419.2335, found 419.2344.

(*S*)-phenethyl 3-(1H-indol-3-yl)-2-(3-phenylpropanamido)propanoate (6e)

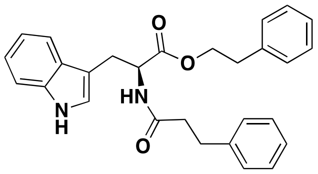

Off-White Solid, Yield: 85%, ^{1}H NMR ($CDCl_3$, 400 MHz): δ 2.32-2.53 (m, 2H), 2.80-3.02 (m, 4H), 3.21 (d, *J* 5.3, 2H), 4.16-4.35 (m, 2H), 4.92-4.95 (m, 1H), 5.88 (d, *J* 7.9, 1H), 6.53 (d, *J* 2.3, 1H), 7.07 (ddd, *J* 8.0, 7.0, 1.0, 1H), 7.12-7.34 (m, 12H), 7.37 (d, *J* 7.9, 1H), 7.96 (br s, 1H), ^{13}C NMR ($CDCl_3$, 100 MHz): δ 27.6, 31.4, 34.9, 38.2, 52.9, 65.9, 109.9, 111.2, 118.6, 119.7, 122.2, 122.7, 126.2, 126.7, 127.7, 128.4, 128.5, 128.6, 128.9, 135.9, 137.6, 140.7, 171.5, 171.7; IR (ATR): υ_{max} /cm^{-1} (neat) 1727, 1656 (C=O): LRMS[a]: calcd for $C_{28}H_{29}N_2O_3$ $(M+H)^+$ 441.2, found 441.0; HRMS (ESI): calcd for $C_{28}H_{29}N_2O_3$ $(M+H)^+$ 441.2179, found 441.2185.

General Procedure for preparation of Tryptamine sulfonamide series.

Tryptamine (1 eq.) was dissolved in anhydrous dichloromethane. Triethylamine (1.1 eq.) was added followed by the corresponding sulfonyl chloride (1 eq.). The reaction was stirred at room temperature until the reaction was deemed complete as determined by the disappearance of the starting materials by TLC (1-12h). The reaction was dissolved in dichloromethane and washed with brine. The layers were separated and the organic layer dried over sodium sulphate. This was filtered and the solvent removed *in vacuo*. The residue placed onto a column of silica gel where the product was eluted with dichloromethane: ethyl acetate (1:0 to 9:1). The solvent was removed *in vacuo* to afford the product.

N-(2-(1H-indol-3-yl)ethyl)benzenesulfonamide (7a)

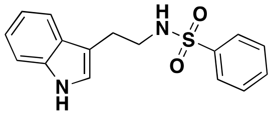

White solid, Yield: 49%, ^{1}H-NMR ($CDCl_3$, 400 MHz) δ 2.94 (t, *J* 6.7, 2H). 3.30 (q, *J* 6.5, 2H), 4.74 (t, *J* 5.7, 1H), 6.94 (d, *J* 2.2, 1H), 7.10 (ddd, *J* 1.0, 7.2, 8.0, 1H), 7.21 (ddd, *J* 1.0, 7.1, 8.1, 1H), 7.36 (d, *J* 8.2, 1H,), 7.45 (m, 3H), 7.55 (tt, *J* 7.4, 2.0, 1H), 7.79 (dd, *J* 1.3, 8.4, 2H,), 8.18 (bs, 1H); ^{13}C-NMR (CD_3OD, 100 MHz) δ 25.5, 43.5, 110.8, 111.1, 117.6, 118.2, 120.9, 122.2, 126.5, 127.1, 128.7, 132.1, 136.7, 140.6; LCMS[a]: calcd for $C_{16}H_{17}N_2O_2S$ $(M+H)^+$ 301.1, found 301.4, R_t = 4.2 min; HRMS: calcd for $C_{16}H_{17}N_2O_2S$ $(M+H)^+$ 301.1011, found 301.1018.

N-(2-(1H-indol-3-yl)ethyl)-1-phenylmethanesulfonamide (7b)

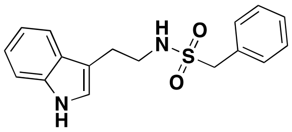

White solid, Yield: 45%, ^{1}H-NMR (400 MHz, $CDCl_3$) δ 2.89 (t, *J* 6.5, 2H,). 3.23 (q, *J* 6.4, 2H,), 4.08 (s, 2H), 6.90 (d, *J* 2.3, 1H), 7.07 (ddd, *J* 1.0, 7.2, 8.0, 1H), 7.18-7.12 (m, 6H), 7.30 (d, *J* 8.1, 1H), 7.48 (d, *J* 7.7, 1H), 7.98 (bs, 1H); ^{13}C NMR (100 MHz,$CDCl_3$) δ 26.6, 44.0, 58.8, 111.8, 112.0, 119.0, 120.1, 122.8, 123.1, 127.3, 129.0, 129.2, 129.7, 130.9, 136.9; IR (ATR): υ_{max}/cm^{-1} (neat) 1410, 1390 (S=O); LCMS[a]: calcd for $C_{17}H_{19}N_2O_2S$ $(M+H)^+$ 315.1, found 314.9, R_t = 4.0 min; HRMS: calcd for $C_{17}H_{19}N_2O_2S$ $(M+H)^+$ 315.1167, found 315.1177.

N-(2-(1H-indol-3-yl)ethyl)-4-bromobenzenesulfonamide (7c)

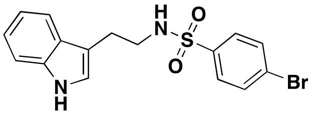

Off-White Solid, Yield: 85%, ^{1}H NMR ($CDCl_3$, 400 MHz): δ 2.95 (t, *J* 6.5, 2H), 3.29 (q, *J* 6.3, 2H), 4.41 (t, *J* 5.7, 1H), 6.97 (d, *J* 1.8, 1H), 7.07 (ddd, *J* 8.2, 7.0, 1.0, 1H), 7.20 (ddd, *J* 8.2, 7.0, 1.0, 1H), 7.34-7.40 (m, 2H), 7.49-7.57 (m, 4H), 8.02 (s, 1H); ^{13}C NMR ($CDCl_3$, 100 MHz): δ 25.5, 43.0, 111.3, 118.4, 119.7, 122.5, 122.6, 126.7, 127.5, 128.5, 132.2, 136.4, 138.7; LRMS[a]: calcd for $C_{16}H_{16}N_2O_2SBr$ $(M+H)^+$ 379.0, found 379.0; HRMS (ESI): calcd for $C_{16}H_{16}N_2O_2SBr$ $(M+H)^+$ 379.0117, found 379.0094.

N-(2-(1H-indol-3-yl)ethyl)-2-bromobenzenesulfonamide (7d)

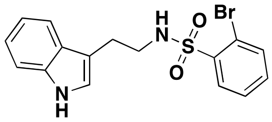

Off-White Solid, Yield: 82%, ^{1}H NMR ($CDCl_3$, 400 MHz): δ 2.95 (t, *J* 6.5, 2H), 3.27-3.31 (m, 2H), 4.41 (br t, *J* 5.6, 1H), 6.96-6.99 (m, 1H), 7.07 (ddd, *J* 8.0, 7.0, 1.0, 1H), 7.2 (ddd, *J* 8.2, 7.0, 1.2, 1H), 7.34-7.41 (m, 2H), 7.49-7.56 (m, 4H), 8.02 (br s, 1H); ^{13}C NMR ($CDCl_3$, 100

MHz): δ 25.5, 43.0, 111.4, 118.4, 119.7, 122.5, 122.6, 126.7, 127.5, 128.5, 132.2, 136.4, 138.8; LRMS[a]: calcd for $C_{16}H_{16}N_2O_2SBr$ $(M+H)^+$ 379.0, found 379.1; HRMS (ESI): calcd for $C_{16}H_{16}N_2O_2SBr$ $(M+H)^+$ 379.0117, found 379.0093.

N-(2-(1H-indol-3-yl)ethyl)-3-nitrobenzenesulfonamide (7e)

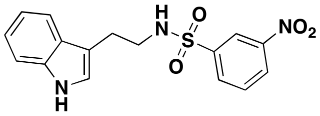

Orange Solid, Yield: 91%, ^1H NMR (DMSO-d[6], 500 MHz): δ 2.96 (t, *J* 7.5, 2H), 3.54-3.59 (m, 2H), 6.94-6.97 (m, 1H), 7.03-7.06 (m, 1H), 7.17 (d, *J* 2.3, 1H), 7.32 (d, *J* 8.1, 1H), 7.56 (d, *J* 7.9, 1H), 7.75 (t, *J* 7.9, 1H), 8.26-8.28 (m, 1H), 8.34-8.36 (m, 1H), 8.66 (t, *J* 1.9, 1H), 8.99 (t, *J* 5.7, 1H), 10.80 (s, 1H); ^{13}C NMR ($CDCl_3$, 125 MHz): δ 25.4, 40.9, 111.8, 112.2, 118.7, 121.4, 122.3, 123.2, 126.2, 127.7, 130.6, 134.1, 136.5, 136.7, 148.2, 164.5; LRMS[b]: calcd for $C_{16}H_{16}N_3O_4S$ $(M+H)^+$ 346.0, found 310.2 (M-36); HRMS: calcd for $C_{16}H_{16}N_3O_4S$ $(M+H)^+$ 346.0862, found 346.0867

N-(2-(1H-indol-3-yl)ethyl)-3-aminobenzenesulfonamide (7f)

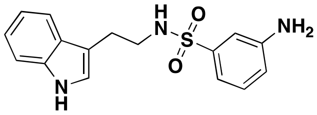

Compound **7e** (0.20g, 0.579 mmol) was dissolved in anhydrous methanol (10 mL) and the reaction flask was flushed with nitrogen. Palladium on carbon (10% w/w on charcoal) (0.020g, 10 wt%) was added to flask and the reaction was placed under an atmosphere of hydrogen using a balloon. The reaction was stirred at ambient temperature for 12 hours. The reaction was flushed with nitrogen and was filtered through Celite™. The solvent was removed *in vacuo* and the residue placed onto a column of flash silica gel where the product was eluted with a gradient of DCM:MeOH in the ratio of 100:0 to 95:5.

Off-white solid, Yield: 83%, ^1H NMR (DMSO-d[6], 500 MHz): δ 2.77 (t, *J* 8.3, 2H), 2.94-2.99 (m, 2H), 5.54 (br s, 2H), 6.72 (ddd, *J* 8.0, 2.4, 0.8, 1H), 6.87-6.89 (m, 1H) 6.92-6.95 (m, 1H), 6.98 (t, *J* 2.0, 1H), 7.01-7.04 (m, 1H), 7.09 (d, *J* 2.3, 1H), 7.16 (t, *J* 7.8, 1H), 7.29 (d, *J* 7.9, 1H), 7.37 (d, *J* 7.9, 1H), 7.51 (t, *J* 5.8, 1H), 10.70 (br s, 1H), ^{13}C NMR ($CDCl_3$, 125 MHz): δ 25.9, 44.1, 111.5,111.6, 111.9, 113.6, 117.5, 118.4, 118.8, 121.4, 123.3, 127.4, 130.0, 136.6, 141.5, 149.5. LRMS[a]: calcd for $C_{16}H_{18}N_3O_2S$ $(M+H)^+$ 316.1, found 316.2; HRMS (ESI): calcd for $C_{16}H_{18}N_3O_2S$ $(M+H)^+$ 316.1120 found 316.1120.

N-(2-(1H-indol-3-yl)ethyl)-4-methoxybenzenesulfonamide (7g)

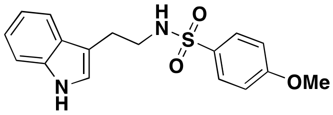

White foam, Yield: 85% ^1H-NMR (400 MHz, $CDCl_3$) δ 2.94 (t, *J* 6.6, 2H), 3.28 (q, *J* 6.5, 2H), 3.85 (s, 3H), 4.59 (bs, 1H), 6.89 (d, *J* 9.0, 2H), 6.97 (d, *J* 2.3, 1H), 7.08 (ddd, *J* 0.9, 7.1, 7.9,

1H), 7.20 (ddd, *J* 1.0, 7.1, 8.1, 1H), 7.36 (d, *J* 8.1, 1H), 7.44 (d, *J* 7.9, 1H), 7.70 (d, *J* 9.0, 2H), 8.16 (bs, 1H); ^{13}C-NMR (100 MHz, $CDCl_3$) δ 25.9, 43.4, 56.0, 111.8, 112.0, 114.6, 118.9, 119.8, 122.6, 123.1, 127.1, 129.5, 131.7, 136.8, 163.2. IR (ATR): υ_{max} /cm^{-1} (neat) 2988, 2902, 1595, 1293; LCMS[a]: calcd for $C_{17}H_{19}N_2O_3S$ $(M+H)^+$ 331.1, found 331.5, R_t = 3.9 min; HRMS: calcd for $C_{17}H_{19}N_2O_3S$ $(M+H)^+$ 331.1116, found 331.1122.

N-(2-(1H-indol-3-yl)ethyl)-2,3-dihydrobenzofuran-6-sulfonamide (7h)

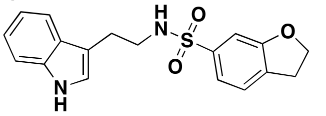

Clear oil, Yield: 21% ^{1}H-NMR (400 MHz, $CDCl_3$) δ 2.96 (t, *J* 6.6, 2H), 3.17 (t, *J* 8.8, 2H), 3.28 (m, 2H), 4.49 (bs, 1H), 4.65 (t, *J* 8.8, 2H), 6.77 (d, *J* 8.3, 1H), 7.00 (d, *J* 2.3, 1H), 7.08 (ddd, *J* 1.0, 7.1, 8.0, 1H), 7.20 (ddd, *J* 1.0, 7.1, 8.1, 1H), 7.37 (d, *J* 8.2, 1H), 7.44 (d, *J* 7.9, 1H), 7.55 (m, 1H), 7.58 (d, *J* 2.0, 1H), 8.16 (bs, 1H); ^{13}C NMR (100 MHz, $CDCl_3$) δ 25.8, 29.4, 43.4, 72.6, 109.7, 111.7, 112.0, 118.9, 119.9, 122.6, 123.1, 124.6, 127.3, 128.7, 129.0, 131.6, 136.8, 164.1; IR (ATR): υ_{max} /cm^{-1} (neat) 1605 (C=C), 1483; LCMS[a]: calcd for $C_{18}H_{19}N_2O_3S$ $(M+H)^+$ 343.1, found 343.5, R_t = 4.4 min; HRMS: calcd for $C_{18}H_{19}N_2O_3S$ $(M+H)^+$ 343.1116, found 343.1123.

N-(2-(1H-indol-3-yl)ethyl)methanesulfonamide (7i)

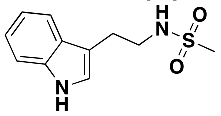

Off-White Solid, Yield: 87%, ^{1}H NMR ($CDCl_3$, 400 MHz): δ 2.82 (s, 3H), 3.05 (t, *J* 6.5, 2H), 3.46, (q, *J* 6.5, 2H), 4.24 (s, 1H), 7.08 (d, *J* 2.2, 1H), 7.12-7.15 (m, 1H), 7.19-7.24 (m, 1H), 7.38 (d, *J* 8.1, 1H), 7.58 (d, *J* 7.9, 1H), 8.07 (s, 1H). ^{13}C NMR ($CDCl_3$, 100 MHz): 26.1, 49.2, 43.2, 111.4, 111.6, 118.5, 119.7, 122.5, 122.6, 126.9, 136.4. LRMS[a]: calcd for $C_{11}H_{15}N_2O_2S$ $(M+H)^+$ 239.0, found 239.1; HRMS (ESI): calcd for $C_{11}H_{15}N_2O_2S$ $(M+H)^+$ 239.0855, found 239.0828.

N-(2-(1H-indol-3-yl)ethyl)thiophene-2-sulfonamide (7j)

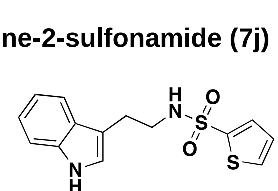

Off-White Solid, Yield: 87%, ^{1}H NMR ($CDCl_3$, 500 MHz): δ 2.95 (t, *J* 6.7, 2H), 3.35 (q, *J* 6.5, 2H), 4.68 (br t, *J* 6.1, 1H), 6.95 (d, *J* 2.3, 1H), 6.98-7.02 (m, 1H), 7.06-7.09 (m, 1H), 7.17-7.20 (m, 1H), 7.34, (d, *J* 8.2, 1H), 7.46 (d, *J* 8.0, 1H), 7.49 (app d, *J* 4.3, 2H), 8.12 (s, 1H); ^{13}C NMR ($CDCl_3$, 125 MHz): δ 25.4, 43.4, 111.3, 111.4, 118.4, 119.5, 122.3, 122.7, 126.9, 127.3, 131.8, 132.1, 136.4, 140.7. LRMS[a]: calcd for $C_{14}H_{15}N_2O_2S_2$ $(M+H)^+$ 307.0, found 307.4; HRMS (ESI): calcd for $C_{14}H_{15}N_2O_2S_2$ $(M+H)^+$ 307.0576, found 307.0568.

N-(2-(1H-indol-3-yl)ethyl)-5-bromothiophene-2-sulfonamide (7k)

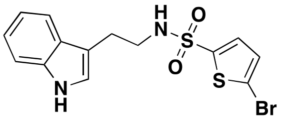

Off-White Solid, Yield: 88%, ^{1}H NMR ($CDCl_3$, 500 MHz): δ 2.97 (t, *J* 6.6, 2H), 3.36 (q, *J* 6.4, 2H), 4.64 (br t, *J* 6.0, 1H), 6.93 (d, *J* 4.0, 1H), 6.98 (d, *J* 2.3, 1H), 7.09 (ddd, *J* 8.0, 7.0, 1.0, 1H), 7.18-7.23 (m, 2H), 7.35 (d, *J* 8.2, 1H), 7.45 (d, *J* 7.9, 1H), 8.08 (br s, 1H); ^{13}C NMR ($CDCl_3$, 125 MHz): δ 25.4, 43.3, 111.3, 111.4, 118.3, 119.6, 119,7, 122.4, 122.7, 126.8, 130.2, 132.0, 136.4, 141.4; LRMS[a]: calcd for $C_{14}H_{14}N_2O_2S_2Br$ $(M+H)^+$ 385.0, found 385.3; HRMS (ESI): calcd for $C_{14}H_{14}N_2O_2S_2Br$ $(M+H)^+$ 384.9681, found 384.9669.

N-(2-(1H-indol-3-yl)ethyl)-5-chlorothiophene-2-sulfonamide (7l)

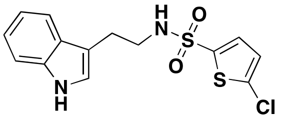

White solid, Yield: 62%, ^{1}H-NMR (400 MHz, $CDCl_3$) δ 2.92 (t, *J* 6.6, 2H). 3.31 (q, *J* 6.4, 2H), 4.44 (t, *J* 5.8, 1H), 6.74 (d, *J* 4.0, 1H), 6.93 (d, *J* 2.3, 1H), 7.03 (td, *J* 7.5, 0.9, 1H), 7.14 (td, *J* 8.1, 1.0, 1H), 7.18 (d, *J* 1.3, 1H), 7.29 (d, *J* 8.1, 1H), 7.39 (d, *J* 7.9, 1H), 7.97 (bs, 1H); ^{13}C NMR (100 MHz, $CDCl_3$) 25.4, 43.3, 111.3, 111.4, 118.4, 119.8, 122.5, 122.7, 126.6, 126.7, 131.4, 136.5, 137.1, 138.6; IR (ATR): υ_{max}/cm^{-1} (neat) 3412, 3242, 1411, 1321. LCMS[a]: calcd for $C_{14}H_{14}ClN_2O_2S_2$ $(M+H)^+$ 341.0, found 340.9, R_t = 4.2 min; HRMS: calcd for $C_{14}H_{14}ClN_2O_2S_2$ $(M+H)^+$ 341.0185, found 341.0194;

N-(2-(1H-indol-3-yl)ethyl)-4-bromo-5-chlorothiophene-2-sulfonamide (7m)

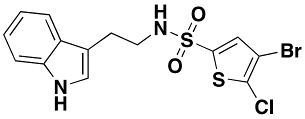

Off-White Solid, Yield: 85%, ^{1}H NMR ($CDCl_3$, 500 MHz): δ 3.01 (t, *J* 6.5, 2H), 3.40 (q, *J* 6.3, 2H), 4.52 (br t, *J* 6.0, 1H), 7.02 (d, *J* 2.3, 1H), 7.10 (ddd, *J* 8.0, 7.0, 1.0, 1H), 7.21 (ddd, 8.0, 7.0, 1.1, 1H), 7.23 (s, 1H), 7.46 (d, *J* 8.2, 1H), 7.45 (d, *J* 7.9, 1H), 8.05 (br s, 1H); ^{13}C NMR ($CDCl_3$, 125 MHz): δ 25.3, 43.3, 110.0, 111.1, 111.4, 118.3, 119.8, 122.6, 126.6, 133.0, 133.3, 136.4, 138.4; LRMS[a]: calcd for $C_{14}H_{13}N_2O_2S_2BrCl$ $(M+H)^+$ 418.9, found 418.8; HRMS (ESI): calcd for $C_{14}H_{13}N_2O_2S_2BrCl$ $(M+H)^+$ 418.9271, found 418.9257.

N-(2-(1H-indol-3-yl)ethyl)-4-bromo-2,5-dichlorothiophene-3-sulfonamide (7n)

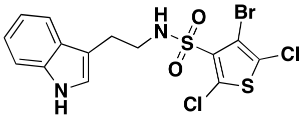

Off-White Solid, Yield: 82%, ^{1}H NMR ($CDCl_3$, 400 MHz): δ 3.00 (t, J 6.5, 2H), 3.40 (q, *J* 6.3, 2H), 4.97 (br t, *J* 5.6, 1H), 7.02 (d, *J* 2.3, 1H), 7.08 (ddd, *J* 8.0, 7.0, 1.0, 1H), 7.19 (ddd, *J* 8.2,

7.1, 1.1, 1H), 7.35 (d, *J* 8.2, 1H), 7.44 (d, *J* 8.2, 1H), 8.06 (s, 1H); ^{13}C NMR ($CDCl_3$, 100 MHz): δ 25.3, 43.0, 108.1, 111.0, 111.4, 118.3, 119.8, 120.4, 122.6, 122.9, 125.4, 126.6, 132.4, 136.6; LRMS: calcd for $C_{14}H_{12}N_2O_2S_2BrCl_2$ $(M+H)^+$ 452.8, found 453.1; HRMS (ESI): calcd for $C_{14}H_{12}N_2O_2S_2BrCl_2$ $(M+H)^+$ 452.8901, found 452.8866.

N-(2-(1H-indol-3-yl)ethyl)-4,5-dibromothiophene-2-sulfonamide (7o)

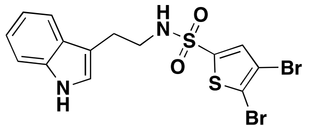

Off-White Solid, Yield: 80%, ^{1}H NMR ($CDCl_3$, 500 MHz): δ 2.99 (t, *J* 6.5, 2H), 3.39 (q, *J* 6.3, 2H), 4.59 (br t, *J* 5.9, 1H), 7.00 (d, *J* 2.3, 1H), 7.07-7.14 (m, 1H), 7.17-7.23 (m, 2H), 7.36 (d, *J* 8.2, 1H), 7.45 (d, *J* 8.0, 1H), 8.05 (s, 1H). ^{13}C NMR ($CDCl_3$, 125 MHz): 25.3, 43.3, 111.1, 111.4, 114.4, 118.2, 118.3, 119.8, 122.6, 122.7, 133.4, 136.4, 141.4; LRMS: calcd for $C_{14}H_{13}N_2O_2S_2Br_2$ $(M+H)^+$ 462.8, found 462.8; HRMS (ESI): calcd for $C_{14}H_{13}N_2O_2S_2Br_2$ $(M+H)^+$ 462.8786, found 462.8750.

N-(2-(1H-indol-3-yl)ethyl)benzo[b]thiophene-2-sulfonamide (7p)

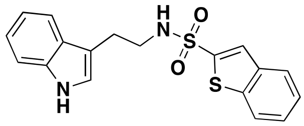

White solid, Yield: 64%, ^{1}H-NMR ($CDCl_3$, 400 MHz) ppm 2.89 (t, *J* 6.6, 2H). 3.34 (q, *J* 6.5, 2H), 4.64 (t, *J* 6.0, 1H), 6.88 (d, *J* 2.3, 1H), 6.91 (ddd, *J* 0.9, 7.0, 7.8, 1H), 7.06 (ddd, *J* 1.1, 7.2, 8.3, 1H), 7.23 (d, *J* 8.2, 1H), 7.36 (m, 3H), 7.66 (s, 1H), 7.71 (m, 2H), 7.95 (bs, 1H); ^{13}C-NMR ($CDCl_3$, 100 MHz) 25.9, 43.9, 111.7, 111.8, 118.8, 120.0, 120.1, 122.8, 123.1, 125.8, 126.0, 127.2, 127.6, 129.6, 136.8, 138.0, 141.2, 142.1; IR (ATR): υ_{max} /cm^{-1} (neat) 1456, 1318; LCMS: calcd for $C_{18}H_{17}N_2O_2S_2$ $(M+H)^+$ 357.1, found 356.9, R_t = 4.3 min; HRMS: calcd for $C_{18}H_{17}N_2O_2S_2$ $(M+H)^+$ 357.0731, found 357.0735.

N-(2-(1H-indol-3-yl)ethyl)-3,5-dimethylisoxazole-4-sulfonamide (7q)

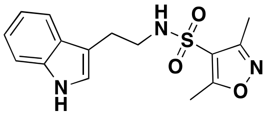

Off-White Solid, Yield: 76%, ^{1}H NMR ($CDCl_3$, 500 MHz): δ 2.15 (s, 3H), 2.47 (s, 3H), 2.99 (t, *J* 6.5, 2H), 3.31 (q, *J* 6.3, 2H), 4.65 (br t, *J* 6.0, 1H), 6.97 (d, *J* 2.3, 1H), 7.06-7.14 (m, 1H), 7.21 (t, *J* 7.7, 1H), 7.36 (d, *J* 8.2, 1H), 7.45 (d, *J* 8.0, 1H), 8.14 (s, 1H); ^{13}C NMR ($CDCl_3$, 125 MHz): δ 10.4, 12.4, 25.4, 42.7, 111.1, 111.5, 115.7, 118.2, 119.8, 122.5, 122.6, 126.6, 136.5, 157.4, 172.9; LRMS[a]: calcd for $C_{15}H_{18}N_3O_3S$ $(M+H)^+$ 320.1, found 320.1; HRMS (ESI): calcd for $C_{15}H_{18}N_3O_3S$ $(M+H)^+$ 320.1070, found 320.1082.

N-(2-(1H-indol-3-yl)ethyl)furan-2-sulfonamide (7r)

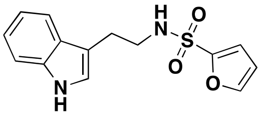

Off-White Solid, Yield: 91%, ^{1}H NMR ($CDCl_3$, 500 MHz): δ 2.98 (t, *J* 6.6, 2H), 3.33 (q, *J* 6.3, 2H), 4.60 (br s, 1H), 6.44 (dd, *J* 2.0, 0.8, 1H), 6.99 (d, *J* 2.1, 1H), 7.09 (ddd, *J* 8.0, 7.0, 1.0, 1H), 7.19 (ddd, *J* 8.2, 7.0, 1.1, 1H), 7.33-7.38 (m, 2H), 7.48 (d, *J* 8.0, 1H), 7.84 (s, 1H), 8.10 (s, 1H) ^{13}C NMR ($CDCl_3$, 125 MHz) δ 25.5, 43.0, 108.0, 111.4, 111.4, 118.4, 119.6, 122.3, 122.6, 126.8, 126.9, 136.4, 144.6, 145.5; LRMS: calcd for $C_{14}H_{15}N_2O_3S$ $(M+H)^+$ 291.1, found 291.0; HRMS (ESI): calcd for $C_{14}H_{15}N_2O_2S$ $(M+H)^+$ 291.0804, found 291.0779.

N-(2-(1H-indol-3-yl)ethyl)-1-methyl-1H-imidazole-4-sulfonamide (7s)

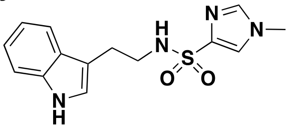

White foam, Yield: 47%, ^{1}H-NMR (DMSO-d^{6}, 400 MHz) δ 2.81 (t, *J* 8.4, 1H), 3.07 (m, 3H), 3.68 (s, 3H), 6.97 (t, *J* 7.9, 1H), 7.06 (t, *J* 8.1, 1H), 7.12 (d, *J* 2.2, 1H), 7.33 (d, *J* 8.1, 1H), 7.41 (d, *J* 7.9, 1H), 7.70 (d, *J* 1.3, 1H), 7.76 (d, *J* 1.0, 1H), 10.80 (s, 1H); ^{13}C NMR (DMSO-d^{6}, 100 MHz) 25.9, 33.7, 43.9, 111.6, 111.7, 117.5, 118.4, 118.6, 121.4, 123.2, 124.4, 127.4, 139.4, 142.4; IR (ATR): υ_{max} /cm^{-1} (neat) 1533,1429; LCMS: calcd for $C_{14}H_{17}N_2O_2S$ $(M+H)^+$ 305.1, found 305.0, R_t = 3.3 min; HRMS: calcd for $C_{14}H_{17}N_4O_2S$ $(M+H)^+$ 305.1067, found 305.1110;

REFERENCES

[1] D. E. Scott, M. T. Ehebauer, T. Pukala, M. Marsh, T. L. Blundell, A. R. Venkitaraman, C. Abell, M. Hyvonen, *ChemBioChem* **2013**, *14*(3), 332-342.

[2] J. Ruiz-Rodriguez, F. Albericio, R. Lavilla, *Chem-Eur J* **2010**, *16*(4), 1124-1127.

[3] J. X. He, W. L. Cody, A. M. Doherty, *J. Org. Chem.* **1995**, *60*(25), 8262-8266.
